# Contribution of Heptose Metabolites and the *cag* Pathogenicity Island to the Activation of Monocytes/Macrophages by *Helicobacter pylori*

**DOI:** 10.3389/fimmu.2021.632154

**Published:** 2021-05-19

**Authors:** Larissa Faass, Saskia C. Stein, Martina Hauke, Madeleine Gapp, Manuel Albanese, Christine Josenhans

**Affiliations:** ^1^ Max von Pettenkofer Institute, Chair for Medical Microbiology and Hygiene, Ludwig Maximilians University Munich, Munich, Germany; ^2^ Institute for Medical Microbiology and Hospital Epidemiology, Hannover Medical School, Hannover, Germany; ^3^ Max von Pettenkofer Institute, Chair for Virology, Ludwig Maximilians University, Munich, Germany; ^4^ Gene Center and Department of Biochemistry, LMU Munich, Munich, Germany; ^5^ German Center of Infection Research (DZIF), Partner site Munich, Munich, Germany; ^6^ DZIF, Partner site Hannover-Braunschweig, Hannover, Germany

**Keywords:** *Helicobacter pylori*, Heptose, macrophage, secretion system, type four secretion system, heptose biosynthesis pathway, lipopolysaccharide

## Abstract

The human gastric pathogen *Helicobacter pylori* activates human epithelial cells by a particular combination of mechanisms, including NOD1 and ALPK1-TIFA activation. These mechanisms are characterized by a strong participation of the bacterial *cag* pathogenicity island, which forms a type IV secretion system (CagT4SS) that enables the bacteria to transport proteins and diverse bacterial metabolites, including DNA, glycans, and cell wall components, into human host cells. Building on previous findings, we sought to determine the contribution of lipopolysaccharide inner core heptose metabolites (ADP-heptose) in the activation of human phagocytic cells by *H. pylori*. Using human monocyte/macrophage-like Thp-1 cells and human primary monocytes and macrophages, we were able to determine that a substantial part of early phagocytic cell activation, including NF-κB activation and IL-8 production, by live *H. pylori* is triggered by bacterial heptose metabolites. This effect was very pronounced in Thp-1 cells exposed to bacterial purified lysates or pure ADP-heptose, in the absence of other bacterial MAMPs, and was significantly reduced upon TIFA knock-down. Pure ADP-heptose on its own was able to strongly activate Thp-1 cells and human primary monocytes/macrophages. Comprehensive transcriptome analysis of Thp-1 cells co-incubated with live *H. pylori* or pure ADP-heptose confirmed a signature of ADP-heptose-dependent transcript activation in monocyte/macrophages. Bacterial enzyme-treated lysates (ETL) and pure ADP-heptose–dependent activation differentiated monocytes into macrophages of predominantly M1 type. In Thp-1 cells, the active CagT4SS was less required for the heptose-induced proinflammatory response than in epithelial cells, while active heptose biosynthesis or pure ADP-heptose was required and sufficient for their early innate response and NF-κB activation. The present data suggest that early activation and maturation of incoming and resident phagocytic cells (monocytes, macrophages) in the *H. pylori*–colonized stomach strongly depend on bacterial LPS inner core heptose metabolites, also with a significant contribution of an active CagT4SS.

## Introduction

The bacterium *Helicobacter pylori* is a chronic human pathogen of major global importance, since about half of the world population carry this bacterial species in their stomach (1). *H. pylori* colonizes primarily the gastric crypts and interacts with the gastric epithelial cell layer. The interaction of *H. pylori* with epithelial cells, primarily of gastric origin, has been very well studied in the past. It is known that *H. pylori* adheres to gastric epithelial cells very specifically, using several different bacterial surface adhesins (bacterial outer membrane proteins), such as AlpA/B, SabA, and BabA/B, HopQ (2–5). On the host (human) side, various receptors, including Lewis antigens (3), cellular integrins, and carcinoembryonic antigen-related cell adhesion molecules (CEACAMs) (6–9) are involved in the interaction. *H. pylori* activates gastric epithelial cells by different innate immune pathways upon intimate cell adherence, involving, among others, TLR2 (10), TLR9 (11, 12), NOD1 (13), and NLRP3 (14, 15) receptors and the newly described ALPK1-TIFA dependent pathway (16–18), which lead to the activation of diverse downstream proinflammatory signaling pathways (19, 20). The ALPK1 pathway is activated mainly by bacterial inner core lipopolysaccharide heptose metabolites, heptose-1,7-bisphosphate (HBP) and, predominantly, ADP-glycero-ß-d-manno heptose (ADP-heptose), which have been reported to interact directly with the kinase ALPK1, resulting in TIFAsome formation and NF-κB activation (18, 21, 22). TLR4 and TLR5 cellular receptors, widely present on epithelial cells, appear to be less activated by the bacteria, since the respective bacterial surface molecules (microbe-associated molecular patterns [MAMP]) of *H. pylori*, lipopolysaccharide (LPS/lipid A) and flagellins, have evolved to low activation potential (10, 23–26). Another very well-studied cell interaction module of *H. pylori* with respect to gastric epithelial cells is the *cag* pathogenicity island (*cag*PAI). The presence of the *cag*PAI determines the extent of gastric inflammation and subsequent disease severity in patients (27) and in animal models (28). This large genetic element is located on the *H. pylori* genome in about 70% of all global isolates (29) and encodes a membrane-spanning secretion system of the type IV (CagT4SS) (30–32). The Cag secretion system is expressed by *H. pylori* in the stomach (33, 34) and can translocate various small molecules into gastric epithelial cells, including bacterial DNA (12), the NOD1 innate receptor ligand ieDAP (13) and lipopolysaccharide (LPS) heptose precursors, HBP and ADP-heptose (17, 21). These small molecule metabolites contribute to different extents and at different times to the epithelial cell activation and modulation by *H. pylori* (13, 16, 20, 35, 36). A substantial portion of early NF-κB activation of epithelial cells by *cag*PAI-positive *H. pylori* appears to be mediated by the CagT4SS-mediated transport of inner core heptose metabolites into the gastric epithelial cells (16–18). At least two CagT4SS transported molecules, the oncogene CagA (37–40) and the peptidoglycan metabolite ieDAP (13, 16), provide signals of sustained, late cell activation (16, 41).

Little information is as yet available on the precise molecular mechanisms of interaction and crosstalk of *H. pylori* with human cells of the myeloid lineages, for instance the phagocytic and antigen-presenting cells (14). The colonization niche of *H. pylori* deep in the gastric mucus layer and within the gastric crypts is characterized by the continuous presence and permanent low-level influx of cells of the myeloid and lymphatic lineages. The immigration of phagocytic cell types, for instance, macrophages and neutrophils, is increased during *H. pylori*-induced inflammation (42). The myeloid and lymphoid cell lineages that *H. pylori* can contact in the gastric mucosa comprise antigen-presenting cell types such as macrophages or dendritic cells, which are probing the mucosa-adherent bacteria or migrating into the mucosa in response to inflammation and cytokines. Neutrophils are attracted to the site of infection, once *H. pylori* have activated local proinflammatory signaling, leading to the secretion of chemokines such as IL-8 (42, 43). It is known that NLRP3 plays a role both for IL-1β production by phagocytic cells in response to *H. pylori* and for bacterial suppression by the immune system *in vivo* (14, 15). The bacterial *cag*PAI is known to be important for the interaction of macrophages with *H. pylori*, even in the absence of TLR signaling (44). Furthermore, it was reported that *H. pylori* directly interacts with stem cells in the depth of gastric crypts (45, 46).

On the basis of the recent discovery of novel cell activation pathways by *H. pylori*, involving the intracellular activation of the ALPK1-TIFA axis and the formation of TIFAsomes, mediated by the inner core LPS heptose metabolite ADP-heptose (16–18, 21), we have now asked the question, how this specific cellular activation pathway contributes to the early and sustained activation and maturation of human cells of the phagocytic lineage, using the human monocyte-like cell line Thp-1 (47, 48) as a model. We demonstrate here that the *cag*PAI-dependent heptose metabolite-triggered early innate activation contributes to a significant extent to cell activation and maturation of human Thp-1 cells and also activates primary human monocytes and monocyte-derived primary macrophages. We also underline and broaden these results by comprehensive transcriptome analyses of heptose- or bacteria-co-incubated Thp-1 cells.

## Materials and Methods

### Bacterial Strains and Cultivation

*H. pylori* bacteria of different strains and mutants (**Table 1**) were cultured under microaerobic conditions (Anaerocult C sachets by Merck) on blood agar plates (Oxoid Blood Agar Base No.2) including 10% horse blood (Oxoid) and the antibiotics (all purchased from SIGMA) amphotericin B (4 mg/L), polymyxin B (2,500 U/L), vancomycin (10 mg/L), trimethoprim (5 mg/L), chloramphenicol (optional; 5 mg/L), and kanamycin (optional; 10 mg/L). Brain Heart Infusion broth (BHI, Becton-Dickinson) supplemented with 5% horse serum (Thermo Fisher Scientific-Gibco) was used for liquid culture of *H. pylori* strains. Routinely, bacteria were cultured at 37°C for 20 h to 24 h on blood agar plates in anaerobic jars, supplemented with humidified Merck Anaerocult C sachets, or in incubators under microaerobic atmosphere (10% CO_2_, 5% oxygen, 85% nitrogen) and passed to fresh plates at least on every second day. Liquid cultures were incubated under microaerobic atmosphere with shaking until mid-log phase (OD_600_ between 0.5 and 1.0), for the collection of culture supernatants. Enzymatically treated lysates (ETLs), enriched in metabolites, were prepared as in (17).

**Table 1:** Bacterial strains and description.

Strain name	Origin	Description	Reference
*H. pylori* (HP) N6 wt	hpEurope; site of isolation = France	Wild type strain, *cag*PAI-positive	(49)
HP N6 *cagY* (HP0527)	hpEurope	Allelic exchange-insertion mutant (HP0527) *(cagY)* in strain N6	(17)
HP N6 858 (HP0858) *(hldE)*	hpEurope	Allelic exchange-insertion mutant (HP0858) (*hldE*) in strain N6	(17)
HP N6 858 (HP0858) comp	hpEurope	HP0858 gene complementation in *rdxA* locus of HP0858 knockout strain N6	(17)
HP 26695a (88-3887) wt	hpEurope; site of isolation = USA	Wild type strains, *cag*PAI-positive	(50)
HP 26695a Δ*cag*PAI	hpEurope	*cag*PAI complete deletion by allelic exchange in strain HP 88-3887 (plasmid pCJ324)	(17)
HP 26695a *cagA*	hpEurope	*cagA* allelic exchange insertion mutant in strains HP 88-3887	(17)
HP L7 wt	hpAsia2, South Asia, India	Wild type strain, *cag*PAI-positive	(29)
HP J99 wt	hpAfrica1, North America	Wild type strain, *cag*PAI-positive	(51, 52)
HP TAI196 wt	hpEastAsia	Wild type strain, *cag*PAI-positive	(29)
HP Africa2 wt	Africa2	Wild type strain, primary *cag*PAI-negative	(51)
HP Su2 wt	hpNEAfrica, Northeast Africa, Sudan	Wild type strain, *cag*PAI-positive	(29)

### Cell Lines and Culture Conditions

In this study, we used the human monocyte leukemia cell line Thp-1 (ATCC TIB202 (47, 48). Thp-1 cells are widely used as a model for monocyte-macrophage-like cells and can be differentiated into active macrophages, for instance using phorbol-12-myristate-13-acetate (PMA, Sigma-Aldrich) treatment (47). For priming, we applied PMA (50 ng/well (53); for 2 days to Thp-1 cells, then gave them a one-day resting period, and then co-incubated the cells with the bacteria on the fourth day, for 4 h. In addition, we employed the NF-κB reporter cell line Thp1_luc (kindly provided by Karsten Tedin) for quantitating NF-κB-dependent responses, and the NLRP3-deficient Thp-1 cell line derivative Thp-1 dNLRP3def (Invivogen). All cell lines were cultured in RPMI1640 medium (buffered with 20 mM Hepes, Glutamax; Thermo Fisher Scientific - Gibco) supplemented with 10% FCS (PromoCell) and, in the case of Thp1_luc cells, were additionally supplied with 0.5 µg/ml puromycin (except when co-incubated with live bacteria or bacterial products). Cells were routinely cultured in a 5% CO_2_ atmosphere incubator. Co-incubation times for each experimental setting are indicated in the figure captions and in the results.

### Co-Culture of Cells With Live Bacteria or Bacterial Products

Co-incubation experiments cells with Thp-1 cells were conducted in either 96-, 24- or 6-well cell culture plates (Greiner BioOne). Thp-1 cells were seeded into fresh RPMI1640 medium 1 h prior to co-incubation with bacteria. Exponentially growing *H. pylori* were harvested from plates and, *via* OD_600_ quantitation, adjusted to the respective multiplicities of infection (MOI) of live bacteria (5, 10, or 25, as detailed for each experiment in the results and respective figures) in RPMI1640 medium containing 10% FCS. Subsequently, macrophages were co-incubated with various bacterial strains, and cell interaction was synchronized using centrifugation (300 × g, 5 min at RT). We chose different co-incubation periods for each type of experiment: For the NF-κB luciferase reporter cell assay, co-incubation was carried out for 4 h. In the case of intended RNA-isolation, cells were co-incubated with bacteria or bacterial products for 8 h. For ELISA cytokine measurements, co-incubations were conducted for 20 to 22 h, before we harvested the cleared cell culture supernatants. Mock-infected cells were used as negative control in each experiment, and Pam3Cys-SK4 (PAMCys, a canonical TLR2 ligand; stock solution of 20 ng/µl) (Invivogen), adjusted to cell number and medium volume in each well plate (standard dilution of 20 ng/50 µl of medium), was co-incubated with cells in each experiment as positive control. In case of comparisons between experiments of the same type, PAMCys coincubation was also used as a normalization control. For further analysis of co-incubated cells *via* RNA-based methods or ELISA, cell pellets and supernatants were harvested and the latter cleared by centrifugation (13,000 × g, 2 min, RT).

Cells were co-incubated with bacterial enzyme-treated lysates (ETLs) at different amounts (volumes between 1 and 50 µl), normalized to bacterial numbers and culture volumes, prepared as outlined in (17). For 96-well co-incubations, we usually used 2.5 µl of bacterial ETL per well (50 µl volume). In 24-well plates, 50 µl of bacterial ETL per 1 ml culture volume were added. By titration in Thp_luc cells, we estimated that 2.5 µl of ETL contained approximately 2.5 µM active heptose metabolites, since the activation was equivalent. Cells were also challenged with pure ADP-heptose (J&K, China) and Pam3Cys-SK4 (Invivogen; activation positive control and normalization control) at concentrations indicated in the results text and figure legends, respectively. To control for cell death, e.g. by pyroptosis, cells during the co-incubation period of 20 h were subjected to a fluorescent live-dead cytotoxicity assay according to the manufacturer’s instructions (CellTiter-Blue Cell Viability Assay; Promega). The results for the different control and co-incubation conditions (bacteria, ADP-heptose) did not show significant signs of cell-death and were not different from the mock co-incubated control condition.

### Cytokine Measurements

We quantitated the following cytokines released into cell supernatants using ELISA measurements: human IL-8 (BD OptEIA set #555244), human IL-1β (BD OptEIA set #557953), human IL-10 (DuoSet ELISA R&D #DY217B-05), human IL-6 (BD OptEIA set #555220), human CCL4 (DuoSet ELISA R&D Systems #DY271-05), human IFN-*γ* (BD OptEIA set #555142). Cytokine secretion into cell supernatants was quantitated using the above commercial ELISA sets and included standard dilutions according to the manufacturer’s instructions. Appropriate dilutions of the cell supernatants for each assay were determined by suitable pre-testing.

### Luciferase Quantitation in Reporter Cells

To quantitate luciferase in NF-κB reporter cells, the Steady-Glo Luciferase Assay System (Promega) was used. Briefly, the lysis buffer-substrate mixture of the system was added to each well and the lysis was allowed to proceed as recommended by the manufacturer’s instructions [see also (54)]. Lysed cells were analyzed for photon counts within 10 min after the lysis using a Clariostar multi-well reader (BMG Labtech) in luminescence mode (acquisition of photons for 10 s, no filter); output is quantitated as counts per second.

### RNA Isolation, cDNA Synthesis and Transcriptome Sequencing (RNA-Seq)

RNA was prepared from Thp-1 cells (monocyte/macrophages) grown in 6-well plates (ca. 2 × 10^6^ cells per well) under co-incubation conditions with *H. pylori* N6 wild type and N6 858 *hldE* mutant (both co-incubated at MOI = 10) and with pure ADP-heptose (at a concentration of 5 µM) for 8 h.

In brief, cell pellets were collected after scraping and rinsing the cells from the plates by centrifugation at 22,000 × g, 1 min, at room temperature. Cell pellets were shock-frozen in liquid nitrogen and stored at −80°C. Total RNA was prepared from each cell pellet using a modified RNeasy spin column protocol (Qiagen, Hilden, Germany) after mechanical lysis in a Fastprep bead-beater (MP Biomedicals Inc., Santa Ana, CA, USA), at power setting 6 for 45 sec. The isolated RNA was quantitated and quality-assessed by photometric measurement, on agarose gels and by tape station (Agilent, Tape Station 4200, RNA nano kit, Agilent) quality controls. All RNAs were treated once with DNaseI (TURBO RNAse-free DNA removal kit; Ambion) according to the manufacturer’s protocol. PCR controls for human GAPDH were performed on each RNA sample pre- and postDNAse treatment to clarify that residual DNA had been removed successfully.

cDNA was routinely synthesized from 1 µg of total RNA, using a combination of random hexamer primers and oligo-dT T12-T18 primers (Invitrogen) and Superscript III reverse transcriptase (Invitrogen) at 42°C for 2 h.

Transcriptome sequencing and analysis was performed after careful quality control of each sample using Agilent tape station and Agilent Bioanalyzer, from 1 µg of total RNA of each sample, using the Illumina NextSeq 500 platform and enrichment of messenger RNAs using poly-T primers before library preparation (non-stranded libraries). The sequencing process was set for a read length of 50 bp on average in each cycle. The output primary raw reads were quality-filtered and trimmed (removal of primer sequences and barcoding) before further analysis.

### Bioinformatics Analysis of RNA-Seq Data

Un-paired fastq files containing trimmed and quality-filtered reads of 50 bp on average for each experimental condition were collected from the sequencing platform (Illumina). For further transcriptome analysis, quality-filtered, trimmed reads were further processed *via* the CLC Genomics Workbench Version 10.1.1 or a higher version (QIAGEN Aarhus A/S). The sample size for all samples was defined to be an equal 2,000,000 reads, and all samples were downsampled (reproducible random setting for downsampling) to this read number. Subsequently, all remaining reads after downsampling were mapped against the human reference genome hg18 (*Homo sapiens* reference genome, ncbi database) in gene track mode (no transcript variants were recorded or analyzed). Read alignment settings were as follows (default settings):

- Mismatch cost = 2
- Insertion cost = 3
- Deletion cost = 3
- Length fraction = 0.8
- Similarity fraction = 0.8
- Strand specific = both
- Maximum number of hits for a read = 10

Expression level options in the RNA-Seq module of CLC Genomics Workbench were set as follows: expression value = Total count; calculate RPKM for genes without considering differential transcripts. Quality control parameters from the sequencing and genome mapping were recorded and compared for all samples according to (**Table 2**). All samples displayed uniform and high-quality control parameters.

**Table 2:** Quality control parameters of comprehensive transcriptome sequencing (RNA-Seq).

Parameters		(N) Thp-1 mock	(O) Thp-1 + N6 wt MOI10	(Q) Thp-1 + N6 858 MOI10	(Z) Thp-1 mock	(A1) Thp-1 + ADP-heptose 5 µM	(A2) Thp-1 mock	(B2) Thp-1 + ADP-heptose 5 µM
Mapping statistics	Reads mapped (%)	81.85	81.88	80.87	83.99	82.23	82.55	82.03
	Reads not mapped (%)	18.15	18.12	19.13	16.01	17.77	17.45	17.97
Fragment counting	Unique (%)	79.12	79.25	78.1	81.47	79.52	79.83	79.27
	Non-specifically (%)	2.73	2.61	2.77	2.52	2.71	2.72	2.76
Counted fragments by type	Intergenic (%)	11.17	10.79	11.65	9.56	10.26	7.72	8.03

RPKM (reads per kilo base [gene length] per million [read count]) values were used for the expression level analysis and further comparisons between samples, as implemented in CLC Genomics Workbench. Differential expressions of all conditions were calculated for all conditions with mock samples as the reference condition, which served as control group. For Venn diagram generation of comparisons between conditions, differential expression values with an absolute fold-change higher than two, four or eight, as indicated in the respective figures and tables, were taken into consideration. The full results of differentially expressed transcripts are shown in **Supplementary Tables S1** and **S2**. The complete transcriptome results are accessible under project no. PRJNA685657 at ncbi.

For protein network analysis from the resulting transcriptome data, the STRING database [https://string–db.org/, (55)] was used. Highly differentially expressed genes overlapping between different samples (threshold of two-fold, four-fold or eight-fold regulated) were uploaded into the platform. Network analysis was performed with a minimum required interaction score of medium confidence (0.004), and only query proteins were allowed to be shown as maximum number of interactions. An absolute fold-change of four-fold or eight-fold regulated transcripts (up- or down-regulated) was used as a threshold to provide a selection of regulated transcripts for further analysis and visualization in STRING as shown in the figures (**Figure 3**; **Supplementary Figure S6**; ball diagrams).

The following STRING settings were used: Proteins with Values/Ranks: minimum required interaction score: medium confidence (0.004); maximum number of interactions to show: 1^st^ shell: none/query proteins only; 2^nd^ shell: none. The level of confidence and the maximum number of interacting proteins were set at 0.4 and 5, respectively.

### Quantitative Real-Time PCR

We performed quantitative (q)RT-PCR as described previously (17). Briefly, (q)RT-PCR was performed on pretested amounts of cDNA specific for each transcript (between 0.5 and 2.5 µl per reaction) using gene-specific primer pairs for human genes (Qiagen Quantitect primer set; primers see **Table 3**), ultrapure water and SYBR Green Master Mix (Qiagen). We performed the reactions in a BioRad CFX96 real-time PCR system (BioRad, Hercules, USA). Quantitation of specific mRNA transcripts in the samples was carried out in technical triplicates. Standards for quantitation in each run stemmed from gene-specific PCR products amplified with the same primers. Results were equalized to 0.5 µl cDNA input and normalized to human GAPDH transcript of each condition, using the Hs_GAPDH transcript amounts from mock-co-incubated cells as reference condition. The MiQE quality settings applied for each qPCR were as described in (56).

**Table 3:** qPCR primers for human genes (Quantitect or RT2—Qiagen).

Name	Gene Symbol	Cat. No
Hs_CXCL8_1_SG	CXCL8	QT00000322
Hs_IL1B_1_SG	IL1B	QT00021385
Hs_ALPK1_1_SG	ALPK1	QT00100842
Hs_CCL4_1_SG	CCL4	QT01008070
Hs_TLR2_1_SG	TLR2	QT00236131
Hs_NLRP3_2_SG	NLRP3	QT01666343
Hs_GAPDH_2_SG	GAPDH	QT01192646
Hs_NOD1_1_SG	NOD1	QT00054082
Hs_TIFA_1_SG	TIFA	QT00212779
Hs_IRF3_1_SG	IRF3	QT00012866
Hs_IRF7 (RT2)	IRF7	PPH02014F (RT2)

### Isolation of Primary Human PBMCs and CD14+ Cells

Human PBMCs were isolated from human blood using negative depletion *via* the MACsPrep PBMC Isolation Kit (negative selection) from 40 ml of whole, serum-depleted anticoagulated blood from healthy donors obtained anonymously from a commercial blood bank. For the isolation of primary CD14^+^ blood monocytes from PBMCs or directly from whole blood/plasma apheresis specimens of anonymous healthy human donors (blood bank), human CD14+ beads (Miltenyi Biotech, Germany), were used in a positive selection protocol as described in the manufacturer’s manual. Alternatively, the selection of primary monocytes from PBMC by plate adherence protocol for monocyte enrichment was used according to (57). After isolation, the monocytes were rested for 24 h in fresh RPMI 1640 supplemented with penicillin/streptomycin and 10% FCS, and then co-incubated with pure ADP-heptose for 20 h at various concentrations indicated in the figure legend. Alternatively, CD14+ monocytes were differentiated with macrophage-colony-stimulating factor (human M-CSF; Peprotech, Germany) for seven days, with one medium change in between. At the end of the differentiation period, cells were visually inspected to be positive for phenotypic maturation, well adherence and colony formation, the medium was changed again to fresh medium (without penicillin-streptomycin and M-CSF) and cells were co-incubated with pure ADP-heptose, PAMCys (positive control for activation), live *H. pylori* bacteria or bacterial treated lysates (ETLs), as indicated in the figure legend, for 4 h. Subsequently to the co-incubations, cell supernatants were collected for cytokine measurements, and cell pellets were collected for RNA isolation. Monocyte purity was verified as follows: collected monocytes were washed once with PBS and resuspended in staining solution (50 µl), consisting of FACS buffer (PBS, 1% FBS, 2 mM EDTA) and α-CD14 Ab (PE clone: M5E2 BD; 555398), and incubated for 20 min at 4°C. Cells were again washed and resuspended in 100 µl FACS buffer. Finally, stained cell suspensions were analyzed in a BD FACS Lyric (Becton, Dickinson and Company).

### siRNA Knock-Down in Thp-1 Cells

For siRNA knock-down of human TIFA transcript in Thp-1 cells, we used the Flexitube Qiagen siRNA Assays, containing four different validated siRNA variants against human TIFA (Flexitube GeneSolution: Hs_TIFA-6, Hs_TIFA-7, Hs_TIFA-8, Hs_TIFA-9, each at 10 µM stock concentration), according to the manufacturer’s instructions. Thp-1 cells (2 × 10^5^ per well) were transfected with siRNAs using the LONZA nucleofector kit SG (nucleofector 4D), with 1.25 µM of siRNA Mix for each siRNA per well (5 µM total of siRNA per well if all were combined, in strip well transfection cuvettes). Immediately after nucleofection, all cells from each cuvette well were resuspended in 1 ml of fresh medium (RPMI1640, 10% FCS) per well, each seeded in 24 well plates, and let acclimatize for 72 h. For each experiment, transfection mix for mock transfection (transfection agent without RNA) and Allstars Negative Control siRNAs Mix (Qiagen) were transfected in separate wells alongside, as negative controls for the knock-down. After 72 h, the cells were again incubated in fresh medium and subsequently incubated in the presence of pure ADP-heptose (5 µM per well), as indicated in the figure captions. At the end of the co-incubation period, cells were gently scraped off the wells, collected including their supernatants, which were then harvested by centrifugation for cytokine ELISA. Cells recovered in the pellets by centrifugation from the supernatants were immediately resuspended in RNA-Later (Agilent) for undamaged RNA isolation. RNA was subsequently isolated from each cell pellet as described above, quality-tested, and used to verify the specific knock-down by qRT-PCR for TIFA transcript (normalized in each condition to Hs_GAPDH transcript). For luciferase detection after siRNA transfection, Thp1_luc cells (2 × 10^5^), containing the NF-κB luciferase reporter, were transfected with siRNA or mock as described above in one strip well, then each transfection well was distributed further into 5 wells of a 96-well plate, in 100 µl of medium (final cell count per well of ca. 4 × 10^4^). The cells were again kept for 72 h for expressing the siRNA. Subsequently, medium was changed to 50 µl fresh RPMI, the cells were co-incubated with pure ADP-heptose for 4 h and then subjected to lysis and luciferase measurement (Promega Steady-Glo firefly luciferase substrate).

## Results

### Live *H. pylori* Activates Thp-1 Monocyte/Macrophage Cells, Dependent on Active Heptose Biosynthesis

**Figure 1:**
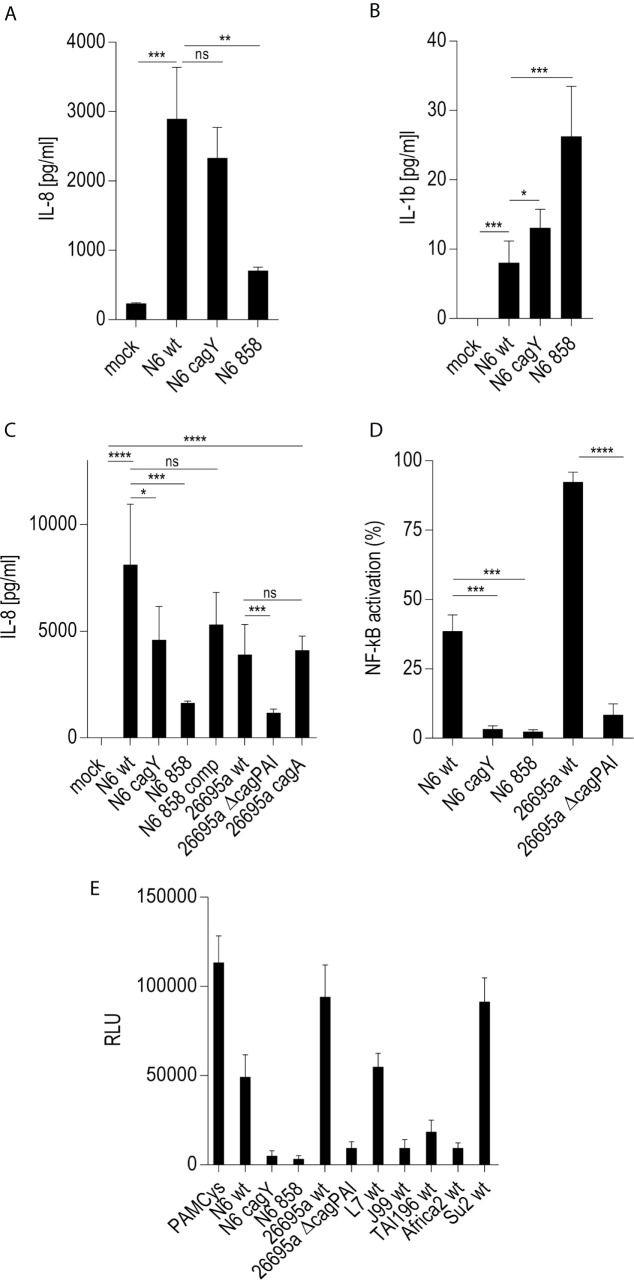
Significant role of LPS inner core heptose biosynthesis and the *cag*PAI in co-incubated Thp-1 cells activated by *H. pylori* or its isogenic mutants. In **(A)**, the IL-8 secretion by Thp-1 cells co-cultured with strain N6 wild type (N6 wt) and isogenic *cagY* and *hldE* (HP0858 – 858) mutants as a marker for cell activation is shown (20 h co-incubation, MOI = 5, no pre-activation). **(B)** low IL-1β secretion in Thp-1 cells co-incubated with the same set of strains at MOI = 10, 20 h (no IL-1β was detected at MOI 5). **(C)** comparison of IL-8 secretion in Thp-1 cells co-incubated with two different *H. pylori* strains (N6 and 26695a) and their isogenic mutants (MOI = 25, 21 h). **(D)** Isolated quantitation of NF-κB activation using Thp1_luc cells, containing firefly luciferase gene under the control of a high-affinity NF-κB promoter (Methods). Thp1_luc cells were co-incubated for 4 h with N6 and 26695a strains and their respective mutants (MOI = 10) as indicated. The values are shown as relative luminescence values, normalized to the activation by heptose-independent, reference substance PAMCys (TLR2 agonist; 20 ng/50 µl), which was set to 100%. **(E)** Quantitation of Thp1_luc NF-κB activation by various *H. pylori* strains (MOI = 10) shown as arbitrary luminescence units (RLU). Comparative values of activation by the PAMCys reference are indicated. One representative independent biological experiment out of three is depicted in each panel. Statistical significance of differences was calculated using unpaired student’s *t*-test. Significant p values: ****p < 0.0001; ***p < 0.001; **p < 0.01; *p < 0.05; ns is non significant.

On the basis of previous results using human epithelial cells (17, 18) we wanted to verify, whether live *H. pylori* bacteria activate phagocyte-like cells using similar pathways or metabolites. We used Thp-1 cells as a model. Thp-1 is a human monocyte leukemia cell line that differentiates into macrophages upon phorbol-12-myristate-13-acetate (PMA) treatment (47). Thp-1 cells have been used extensively to study monocyte/macrophage signaling pathways, responses and phagocytosis mechanisms (47, 58). In these assays, we also wanted to assess at the same time, whether the bacteria engage predominantly TLR-mediated (15, 25, 26), NLR-mediated (13–15), or (ADP-)heptose metabolite-mediated recognition (17, 21, 22), in order to activate phagocytic cells at early time points. We initially co-incubated Thp-1 cells with live *H. pylori* bacteria for different time periods. We used different *H. pylori* strains and mutants, including mutants deficient in the CagT4SS and mutants deficient in the LPS-heptose biosynthesis pathway (17) for the cell activation. Thp-1 monocyte-like cells were alternatively pretreated (primed with PMA) to differentiate them into an adherent, macrophage-like cell type, or not pretreated, before co-incubating them with live *H. pylori*. IL-8 cytokine release into the cell supernatant was quantitated, as an outcome of either IL-1/TLR receptor pathway activation or as a marker for LPS core heptose activation in the cells. In addition, IL-1β secretion by the cells and transcript amounts were determined, as IL-1β is a signature cytokine for combined IL-1/TLR and NLR pathway and inflammasome activation. When we co-cultured non-primed Thp-1 cells with live *H. pylori* bacteria for at least 8 h (20 h time point shown (**Figure 1A** **)**, the cells started to morphologically differentiate into an adherent cell type, corresponding to a macrophage-like cell morphology. IL-8 cytokine secretion was determined by ELISA. The amount of IL-8 released at 20 h post-co-incubation was partially associated with an active *cag*PAI and strongly correlated with active LPS heptose biosynthesis (**Figure 1**). Cell activation by heptose (*hldE*) mutants in this setting was significantly lower than by parental wild type bacteria, but significantly higher than for mock-co-incubated cells. Likewise, IL-1β release upon *H. pylori* co-culture was influenced by heptose biosynthesis (**Figure 1**). While the non-differentiated Thp-1 cells did not produce detectable amounts of IL-1β in response to *H. pylori* at early time points below 8 h (not shown), they released low amounts of IL-1β at 20 h co-incubation, which was significantly higher than under the mock-co-incubated conditions (**Figure 1**). IL-1β release was less associated with the CagT4SS, and *cag*PAI and heptose (*hldE*) mutants still provoked a significantly higher IL-1β release by Thp-1 cells than mock conditions. In the setting without prior cell priming, the differential proinflammatory response of phagocyte-like cells to *H. pylori* was exquisitely MOI-dependent (**Supplementary Figure S2**), although the heptose mutants activated the cells significantly less than wild type bacteria at all MOIs up to 25. Various wild type strains at the same MOI activated the Thp-1 cells to a significantly different extent at 20 h post co-incubation (p.c.) (**Figures 1C–E** **)**. *cagA* mutants did not show a significant difference in cell activation in comparison to wild type bacteria in any of the settings, while Δ*cag*PAI mutants or T4SS-inactive (*cagY*) mutants showed a significantly reduced activation potential (IL-8 release) as compared to the parental wild type strain (**Figure 1**). It is known that bacterial heptose metabolites such as ADP-heptose can activate cellular NF-κB at early time points (16, 17, 21, 59), which, in gastric epithelial cells co-incubated with live *H. pylori*, is mediated by an active CagT4SS (17). Subsequent results that we gathered from co-incubation experiments with a Thp1_luc NF-κB luciferase reporter cell line, which allows to specifically quantitate NF-κB activation (54), underlined that, also in the monocyte/macrophage cells, the early NF-κB response induced by live *H. pylori* is strongly heptose- and CagT4SS-dependent; heptose biosynthesis mutants (*hldE*), Δ*cag*PAI (complete T4SS-deficient) and *cagY* (T4SS functionally deficient) mutants were strongly impaired in inducing NF-κB activation (**Figure 1D** **)**. In addition, we determined a remarkable diversity between different *H. pylori* wild type strains to induce NF-κB specifically in the non-preprimed luciferase reporter cells (**Figure 1**). Live *H. pylori* also activated NF-κB in a strongly CagT4SS- and heptose-dependent manner (**Figure 1**). This effect paralleled the cytokine measurements, but clearly showed a more distinct difference between wild type and the *cag*PAI (*cagY*) or *hldE* mutant (**Figure 1**). *hldE*-complemented bacteria recovered the cell activation properties on Thp-1 cells (**Figure 1E**). In an alternative set-up, we also primed the Thp-1 cells prior to bacterial co-incubation using PMA (47, 53) or *Escherichia coli* LPS (not shown). While co-incubation with live *H. pylori* activated the pre-primed cells to produce increased amounts of IL-8 and more IL-1β as compared to mock-coincubation (**Supplementary Figures S1A, B**), both *H. pylori* wild type and all different mutants strongly and uniformly activated the cells. No significant reduction in cytokine release was observed for the CagT4SS mutants or the heptose biosynthesis mutants as compared to wild type bacteria in this setting (**Supplementary Figures S1A, B**). Interestingly, in the primed setting, for IL-1β release, the ADP-heptose-negative *hldE* mutant (HP0858-mut) showed even a significant increase in IL-1β release over the wild type bacteria, similar but stronger than in the non-primed conditions. However, in general IL-1β release in these settings was rather low, with or without PMA activation.

We also addressed the question, whether co-incubation with live *H. pylori* differentiated the Thp-1 monocytes rather to an M1-like (proinflammatory) or M2-like macrophage phenotype (44, 60–62). M1 macrophages tend to produce higher amounts of IL-6 (and IL-8) while M2 macrophages predominantly produce IL-10, less IL-6 or IL-8 (63). M1 tend to upregulate STAT1, STAT2, and IRF5, while M2 rather increase STAT3, STAT6, IRF3, and IRF4 (62). Thp-1 co-incubated with *H. pylori* (at MOI 10) produced copious amounts of IL-8 and, to a lesser extent IL-6 (**Supplementary Figure S3**), but produced no (at 8 h p.c.) or very low (at 20 h p.c.) amounts of IL-10. A trend to differentiation towards a proinflammatory M1 macrophage phenotype was also supported by the transcriptome results (see below), which showed an increased expression of M1-related upstream activator and M1-specific transcription factor genes such as STAT1, STAT2, and AP-1 (64) as well as IRF5 (62), while M2-specific STAT6, IRF3, and IRF4 transcripts were rather downmodulated or not increased (**Supplementary Figures S4A, B**).

### Early Activation of Cellular Signaling by *H. pylori* in Thp-1 Cells Is Directly Modulated by Heptose Metabolites and an Active CagT4SS; Free Heptose and Bacterial ETLs Can Activate in the Absence of an Active T4SS

**Figure 2:**
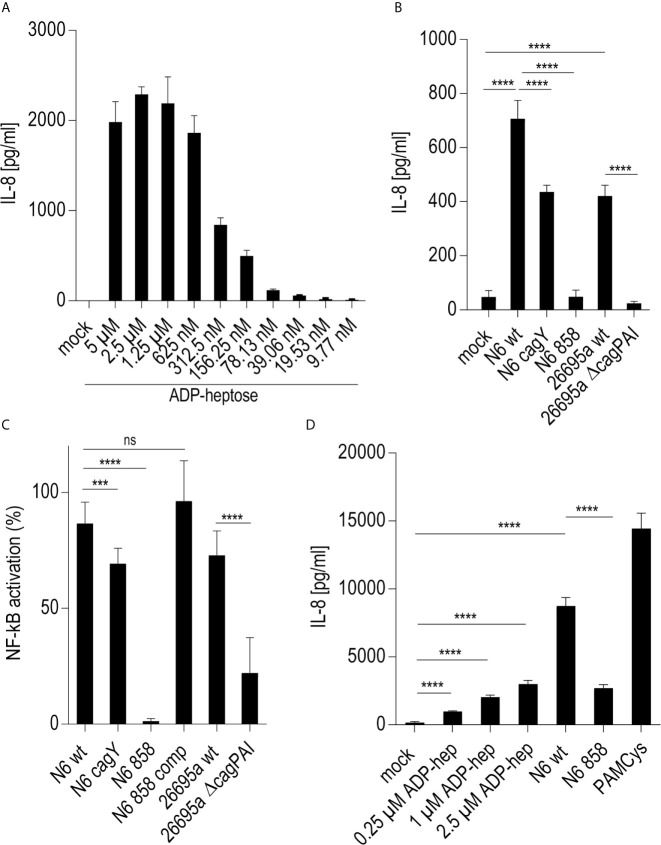
Activation of monocyte/macrophage cell line Thp-1 and primary human macrophages by pure ADP-heptose, enzyme-treated bacterial lysates (ETL), and live *H. pylori*. **(A)** dose-dependent activation of Thp-1 cells by pure ADP-heptose: ADP-heptose in different concentrations as indicated was applied to Thp-1 monocyte/macrophages for 20 h in 24-well plates, and IL-8 secretion was quantitated using ELISA (for comparison, concentration-dependent activation of NF-κB reporter cells Thp1_luc by ADP-heptose is shown in **Supplementary Figure S3A**). **(B)** Thp-1 cells were co-incubated with ETLs of two *H. pylori* strains and their isogenic *cagY*, Δ*cag*PAI, and *hldE* mutants for 20 h in 24-well plates (50 µl of lysates per well added to cells), followed by the quantitation of IL-8 secreted in the cell supernatant by ELISA. **(C)** ETLs prepared from two different *H. pylori* strains including their isogenic heptose mutant (N6 858) and complementant (N6 858 comp), *cagY* and *cag*PAI-deletion mutant ETLs were comparatively tested for NF-κB induction in 96-well format (Thp1_luc cells, 4 h co-incubation, 2.5 µl of lysate/well). Relative values in %, normalized to an independent reference (PAMCys) which was set to 100%, are depicted. *cagY* ETL led only to a mild reduction of NF-κB compared to wt ETL, while *cag*PAI mutant ETL was associated with a strong reduction of activation. ETLs or pure ADP-heptose alone did not induce significantly higher IL-1β secretion than mock-co-incubated cells under the tested conditions, while PAMCys induced IL-1β (not shown). No pre-activation or priming was used in these assays. One of at least three independent biological experiments is shown in each panel. **(D)** Primary human monocyte-derived macrophages (hMDMs) were treated with pure ADP-heptose in different concentrations (hep; as indicated), with PAMCys (400 ng/ml) and with live bacteria (MOI 5; N6 wt is *H. pylori* N6; N6 858 is isogenic *H. pylori hldE* mutant deficient in LPS core heptose biosynthesis) in 24-well plates. Cell activation was quantitated by IL-8 ELISA. Primary macrophages were significantly activated by ADP-heptose in a concentration-dependent manner. Biosynthesis-deficient *hldE* mutant activated significantly less than *H. pylori* wild type bacteria. Mean and standard error of six replicates is shown for each condition. Statistical significance of differences in **(A–D)** was calculated using unpaired student’s *t-*test. Significant p values: ****p < 0.0001; ***p < 0.001; ns is non significant.

Since live *H. pylori* bacteria activated monocytes to a macrophage-like phenotype, in a partially *cag*PAI- and heptose-dependent manner, we sought to determine whether the presence of the Cag injection apparatus or an injected molecule, most likely ADP-heptose, was the main contributing factor. We prepared *H. pylori* bacterial supernatants and bacterial enzyme-treated lysates (ETL) (17), enriched in various metabolites including heptoses (21), and comparatively tested pure ADP-heptose. Pure ADP-heptose dose-dependently activated Thp-1 cells to produce high amounts of IL-8 (**Figure 2**), but not IL-1β or IL-10 (not shown), at 20 h p.c. Since cellular NF-κB activation was directly correlated to prior ADP-heptose activation in previous studies of epithelial cells (16, 17, 21), we complemented this approach using the Thp1_luc NF-κB-luciferase reporter cells, which provide a direct and high-throughput detection and quantitation system for NF-κB activation. Pure ADP-heptose directly activated NF-κB in the Thp-1 reporter cells to a high extent at 4 h post-co-incubation and at later time points (not shown), similarly to PAMCys (TLR2 ligand) which we used as normalization and positive control condition (**Supplementary Figure S3A**).

In human epithelial cells, the CagT4SS seems to be required to transport heptose metabolite(s) into the cells when exposed to live bacteria (17). However, free heptoses can activate epithelial cells in the absence of bacteria (21). It was not known before for monocyte/macrophage-like cells, whether bacterial supernatants or bacterial metabolite-enriched lysates are able to activate in the absence of live bacteria. In order to determine, whether the soluble metabolites, including LPS heptose metabolites, produced by *H. pylori* are able to activate Thp-1 cells independently of transfection or a transport system, we prepared bacterial supernatants and ETL lysates (17), from various *H. pylori* strains and mutants. We co-incubated Thp-1 NF-κB luciferase reporter cells with pure ADP-heptose, with selected *H. pylori* ETLs from wild type and mutants, or with bacterial supernatants. Pure, externally added ADP-heptose activated the cells and NF-κB signaling to a high extent and concentration dependently at 4 h post-co-incubation (**Figure 2**, **Supplementary Figure S3A**). Similarly, ETLs from wild type *H. pylori* added to the cell culture medium strongly activated NF-κB (**Figures 2B** **)**. This activation potential in Thp-1 cells was very similar for ETLs derived from wild type strains and CagT4SS-deficient mutants (**Figure 2C**). In contrast, ETLs generated from heptose-negative *hldE* mutants activated NF-κB to a significantly lower extent (**Figure 2**), only slightly more than the activation recorded in mock-co-incubated cells. Bacterial supernatants, heptose-dependently and strain-dependently, also activated Thp-1 luciferase cells, but considerably less than ETLs (**Supplementary Figure S3C**), indicating that the intact bacteria grown in the absence of cells release only low amounts of free heptose metabolites into the medium. Free NOD1 or NOD2 ligands as well as purified bacterial DNA (TLR9 ligand) did not activate the Thp-1 cells in this setting (**Supplementary Figure S4**), indicating that NOD and TLR9 signaling do not play a role to activate Thp-1 cells.

In order to block phagocytic uptake, the cytoskeleton modulator cytochalasin D (CytD) was used in co-incubation experiments. CytD inhibited the induction of IL-8 secretion in Thp-1 cells co-incubated with pure ADP-heptose, however only by about 30% (**Supplementary Figure S3D**). This was also true for PAMCys co-incubation, exemplarily used with the intent to activate the IL-1/TLR (TLR2) response pathway (**Supplementary Figure S3D**). Similarly, *cag*PAI- and heptose-dependent effects of NF-κB induction upon co-incubation with *H. pylori* ETLs or live *H. pylori* in Thp-1 cells were partially, but not completely, inhibited by CytD. We also addressed the question, whether phagocytic uptake in general was influenced by heptose metabolite exposure of the cells. We incubated Thp-1 cells with microbeads in the absence or presence of pure ADP-heptose for 20 h (**Supplementary Figure S4E**). Beads were taken up by the cells and no significant difference in bead uptake between heptose-co-incubated or heptose-free beads was determined (**Supplementary Figure S4E**).

### Primary Human Monocytes and Matured Primary Macrophages Are Activated by Pure ADP-Heptose, and *H. pylori* Heptose Biosynthesis Competence Codetermines Activation Potential

In order to verify that *H. pylori* can activate primary human phagocytes in a heptose-dependent manner, as determined before in co-incubated Thp-1 cells for pure ADP-heptose and bacteria, we isolated primary CD14-positive monocytic cells from human peripheral blood. We co-incubated the purified primary human monocytes directly with pure ADP-heptose, without further maturation, at different concentrations for 20 h (**Supplementary Figure S5**). The outcome clearly demonstrated a significant and concentration-dependent activation of the non-differentiated CD14+ cells by pure ADP-heptose (**Supplementary Figure S5A**), similar to the co-incubated Thp-1 cells. In addition, we differentiated the CD14+ cells to human monocyte-derived macrophages (hMDMs) for seven days by pre-incubation with M-CSF, and then subjected them to pure ADP-heptose at different concentrations (**Supplementary Figure S5B**), or co-cultured them with live bacteria, for 4 h. In the matured hMDMs, we observed as well a significant activating effect of pure ADP-heptose (**Figure 2D**, **Supplementary Figure 5B**). hMDMs co-incubated with live bacterial strains or treated bacterial lysates enriched in metabolites (ETL) showed a clear difference between a heptose-positive wild type strain and an isogenic *hldE-* mutant (**Figure 2D**, **Supplementary Figure 5C**). These activation effects were similar for primary monocytes and differentiated hMDMs obtained from independent donors.

### Regulation of the Genome-Wide Transcriptome in Thp-1 Cells by *H. pylori* Is Strongly Dependent on the LPS Core Heptose Pathway and Is Partially Recapitulated by Pure ADP-Heptose

**Figure 3:**
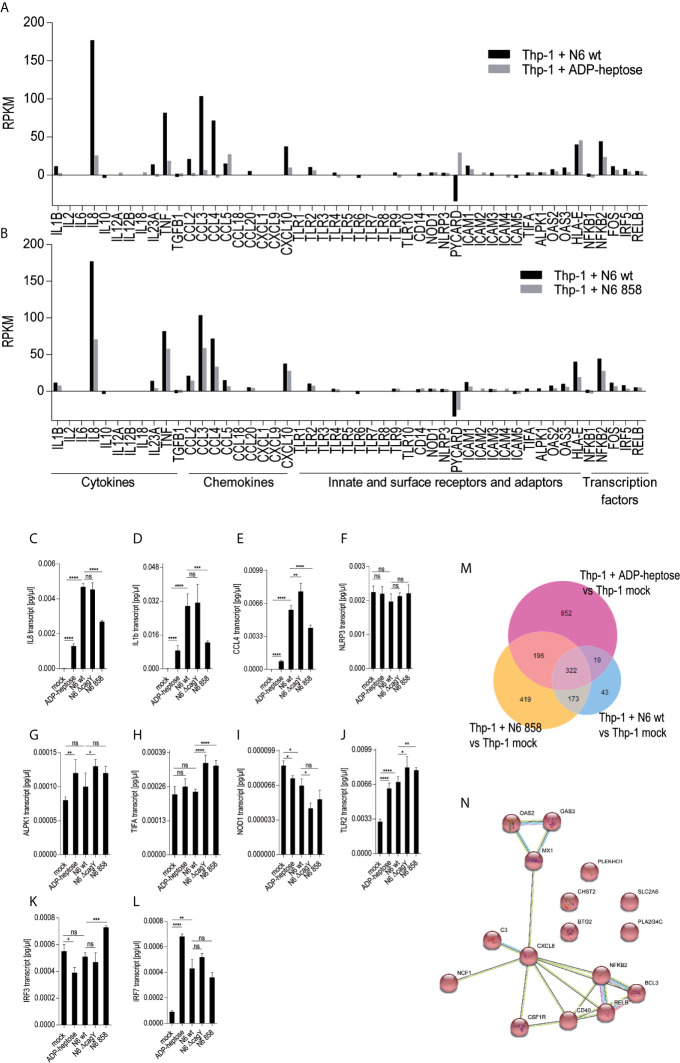
Transcriptional activation of monocyte/macrophage cell Thp-1 by live *H. pylori* and pure ADP-heptose. We performed comprehensive transcriptome assays (RNA-Seq) of cells that were co-incubated for 8 h with *H. pylori* strains and pure ADP-heptose (selected results in **Table 4**, **Supplementary Tables S1, S2**, and full results under project No. PRJNA685657 deposited at ncbi). **(A)** Selected results (panel of innate immune-related genes) of differential transcript amounts (RPKM relative to mock-co-incubated conditions; mock values were subtracted) after 8 h of co-incubation of *H. pylori* N6 wt live bacteria (MOI = 10, black bars) and ADP-heptose (5 µM; grey bars). **(B)** Selected results (panel of innate immune-related genes) of differential transcript amounts (RPKM relative to mock-co-incubated, mock values were subtracted) after 8 h of Thp-1 co-incubation of *H. pylori* N6 wt live bacteria (MOI = 10, black bars) and *hldE* mutant bacteria (MOI = 10; grey bars). Panels **(C–L)** show qPCR results of selected genes by Thp-1 cells co-cultured with live N6 wild type and mutants, also including the *cagY* mutant, which was not included in the RNA-seq analysis. **(C–E)** show qPCR results for downstream activated cytokine genes il8, il1b, and ccl4; **(F–L)** show genes coding for relevant pattern recognition receptors, adaptors, and transcription factors: ALPK1, TIFA, NLRP3, TLR2, NOD1, IRF3, IRF7. Results are quantitated as absolute values [pg/µl], normalized to human GAPDH transcript for each condition. Statistical differences were calculated by unpaired student’s *t*-test. **(M)** Venn diagram comparing the differentially expressed genes between live *H. pylori* N6 wt bacteria *versus* mock-co-incubated (blue circle), live *H. pylori hldE* (HP0858) mutant-co-incubated *versus* mock (orange), as well as ADP-heptose-treated cells *versus* mock (pink circle). The genes contained in the intersection of comparisons (overlap of differentially expressed genes) between wt bacteria-activated and ADP-heptose- activated cells are shown in **Supplementary Table S2**). **(N)** Intersection of Venn diagram of two RNA-Seq experiments of ADP-heptose-co-incubated Thp-1 cells vs. mock-co-incubated, visualized as a pathway map in STRING (all are upregulated transcripts, colored in red). The threshold was set to 8-fold change to visualize a reduced selection of regulated genes. The table view for this comparison is shown in **Table 4** (for all genes regulated above a four-fold cut-off) and in **Supplementary Table S2**. All other comparisons were performed with a threshold of (+/-)2-fold regulated. (For selected extended results see Excel **Supplementary Table S1**). Significant p values for differences shown in **(C–L)** were calculated by unpaired Student’s *t*-test: ****p < 0.0001; ***p < 0.001; **p < 0.01; *p < 0.05; ns is non significant.

In order to obtain a comprehensive overview and compare transcript regulation induced by free ADP-heptose or by live *H. pylori* and its heptose metabolites in Thp-1 cells, we harnessed global transcriptome assays. Initially, we assessed the activity of free ADP-heptose co-incubation on the complete Thp-1 cell transcriptome. For this purpose, non-pre-differentiated Thp-1 cells were treated with free ADP-heptose (2.5 µM) for 8 h, RNA was isolated, and the comprehensive cellular transcriptome was analyzed by deep sequencing (RNA-Seq, Methods). For comparison to this primary core dataset, live *H. pylori* wild type bacteria and isogenic heptose biosynthesis mutants (*hldE*) were also co-incubated with the non-primed Thp-1 cells for 8 h. cDNAs generated from all these settings were then also subjected to transcriptome sequencing and analyzed. In this comprehensive analysis, ADP-heptose-co-incubated cells showed numerous differentially regulated transcripts in comparison to mock-co-incubated, which was directly related to ADP-heptose activation (**Figures 3A, M, N**; **Supplementary Table S1**). These experiments confirmed again that cell transfection was not necessary to provide ADP-heptose access to those cells. Signature transcripts of Thp-1 enhanced by ADP-heptose or live bacteria at the early time points encompassed il-8 (cxcl8), ccl2, ccl3, ccl4, and il-1β, but not il-6 or il-10 (**Figure 3D**, **Table 3** and **Supplementary Tables S1, S2**). We also detected a substantial overlap of transcripts regulated by co-incubation of Thp-1 cells with either pure ADP-heptose or with live bacteria *of H. pylori* wild type (**Figure 3B**, **Table 4** and **Supplementary Table S2**). Dominantly regulated transcripts by cell co-incubation with pure ADP-heptose (cut-off of eight-fold regulated) included a strong upregulation of NF-κB subunit genes for NFKB2 and RELB and downstream IL-8 and complement factor C3. Likewise, transcriptional coactivator BCL3, and OAS2, OAS3 genes involved in innate recognition of double-stranded RNA (65), as well as CD40 and a macrophage glycan transporter gene of unknown specificity, SLC2A6, were strongly upregulated (**Figure 3**, **Table 4**). While about 340 differentially regulated transcripts (**Figure 3M**, **Supplementary Table S2**) overlapped between ADP-heptose-co-incubated cells and *H. pylori* wild type bacteria-exposed cells at a cut-off of two-fold regulated, pure ADP-heptose treatment (ca. 852 transcripts; **Tables 1, 2**) and Cag-positive *H. pylori* wild type bacteria (419 transcripts, **Supplementary Table S2**) also regulated a subset of specific transcripts differentially. Overlapping and distinct transcript regulation was also identified for a comparison of the respective comprehensive transcriptomes, between the genes differentially regulated over mock conditions by the *hldE* mutant and the parental wild type bacteria (**Supplementary Figure S6**, **Supplementary Table S2**). Numerous genes were differentially regulated between *H. pylori* wild type-co-incubated and *H. pylori* heptose-mutant-co-incubated cells (**Figure 3**). As expected, il-8 was among the differentially regulated genes/transcripts between mock and wild type bacteria as well as between ADP-heptose-co-cultured and mock cells, and between *hldE* and wild type bacteria-exposed. Despite the clear differences in transcript regulation between *H. pylori* wild type and *hldE*-mutant co-incubated cells, we also found a substantial overlap of regulation between those two conditions over mock, which were not quite comparable in strength of regulation, but encompassed a high number of similarly up-or downregulated transcripts (**Supplementary Figure S6**; **Supplementary Table S2**). Among the most strikingly differentially regulated transcripts between *H. pylori*-exposed and free heptose-treated cells was the downregulation of PYCARD/ASC transcript for live *H. pylori versus* mock, and the upregulation of the same transcript for the ADP-heptose-treated cells *versus* mock-treated.

**Table 4:** Selected human gene transcripts regulated by ADP-heptose co-incubation of human monocyte/macrophage cells line Thp-1 (cut-off of fourfold regulated in comparison to mock, from two independent experiments [biological replicates]).

Name	Thp-1 ADP-heptose *vs.* Thp-1 mock (A2B2)					Thp-1 + ADP-heptose *vs.* Thp-1 mock (ZA1)				
	Max group mean (RPKM)	Fold change	P-value	FDR p-value	Bonferroni	Max group mean (RPKM)	Fold change	P-value	FDR p-value	Bonferroni
SLC2A6	6.59	92.71	2.13E-06	5.50E-04	0.08	10.85	43.8	6.81E-14	1.01E-11	2.50E-09
OAS3	1.06	39.57	1.82E-07	6.59E-05	6.66E-03	4.1	30.75	0	0	0
PLA2G4C	0.29	31.58	3.48E-04	0.04	1	0.8	25.26	4.10E-10	3.55E-08	1.50E-05
BCL3	3.09	22.37	1.42E-07	5.62E-05	5.22E-03	7.85	26.25	1.11E-16	2.31E-14	4.07E-12
IL8	11.03	22.05	1.64E-07	6.14E-05	6.02E-03	26.84	24.76	2.22E-16	4.50E-14	8.15E-12
CHST2	6.09	20.53	2.24E-05	4.08E-03	0.82	7.19	48.25	5.44E-05	1.46E-03	1
RELB	2.8	18.2	0	0	0	5.11	19.32	0	0	0
C3	0.69	14.01	4.65E-07	1.47E-04	0.02	3.83	17.78	0	0	0
CSF1R	0.45	12.78	1.23E-06	3.40E-04	0.05	2.17	12.2	0	0	0
MX1	1.62	11.1	9.13E-14	1.04E-10	3.35E-09	9.25	38.77	0	0	0
OAS2	0.67	10.57	8.30E-06	1.73E-03	0.3	4.95	21.15	0	0	0
CD40	2.94	9.88	4.67E-10	2.95E-07	1.71E-05	6.06	27.45	0	0	0
NFKB2	19.06	9.26	0	0	0	25.58	14.96	0	0	0
C1orf147	2.1	8.78	3.63E-04	0.04	1	6.21	4.45	1.11E-07	6.28E-06	4.09E-03
NCF1	4.56	8.54	6.22E-15	8.45E-12	2.28E-10	17.92	33.25	0	0	0
BTG2	7.22	8.11	4.57E-07	1.46E-04	0.02	24.38	10.19	0	0	0
PLEKHO1	6.75	8.1	6.22E-15	8.45E-12	2.28E-10	6.53	12.71	2.89E-15	5.02E-13	1.06E-10
FEZ1	1.22	7.83	7.77E-16	1.19E-12	2.85E-11	2.32	5.05	0	0	0
GAS7	0.34	7.73	0	0	0	0.74	7.86	0	0	0
FTHL16	367.92	7.43	0	0	0	997.03	13.9	0	0	0
NCF1B	2.22	7.33	9.22E-08	3.89E-05	3.38E-03	4.6	13.09	1.22E-15	2.27E-13	4.48E-11
RP11-274P12.1	596.02	6.94	0	0	0	1,535.62	17.35	0	0	0
RP4-646B12.2	844.59	6.87	0	0	0	1,637.92	11.85	0	0	0
TNFAIP3	7.66	6.8	0	0	0	13.81	10.28	0	0	0
IFI6	4.43	6.61	1.76E-06	4.59E-04	0.06	38.61	34.82	0	0	0
PARP14	1.08	6.21	2.01E-11	1.64E-08	7.36E-07	5.52	12.39	0	0	0
NFKBIA	47.84	5.94	0	0	0	71.83	10.19	0	0	0
PDGFA	1.59	5.93	8.94E-08	3.81E-05	3.28E-03	0.98	6.58	7.73E-06	2.67E-04	0.28
CYBB	6.78	5.7	0	0	0	5.19	7.62	0	0	0
LIMD2	35.69	4.98	0	0	0	66.45	6.31	0	0	0
TRIM16L	1.61	4.89	3.39E-05	5.67E-03	1	3.62	9.46	3.00E-11	3.08E-09	1.10E-06
AKR1C1	4.08	4.76	2.42E-11	1.85E-08	8.87E-07	6.72	5.74	0	0	0
MSC	8.89	4.65	1.26E-05	2.50E-03	0.46	24.67	24.64	2.41E-14	3.83E-12	8.84E-10
CD44	0.35	4.59	1.57E-06	4.17E-04	0.06	0.57	8.34	2.75E-12	3.29E-10	1.01E-07
CD83	1.76	4.42	1.52E-06	4.11E-04	0.06	2.08	4.58	1.15E-07	6.43E-06	4.21E-03
LPXN	0.62	4.23	6.27E-06	1.39E-03	0.23	2.93	18.45	0	0	0
MT2A	68.46	4.21	7.99E-11	5.53E-08	2.93E-06	172.54	9.53	0	0	0
TFEB	0.65	4.15	2.56E-06	6.49E-04	0.09	1.37	6.73	3.44E-15	5.93E-13	1.26E-10
TYMP	32.11	4.15	0	0	0	63.05	12.66	0	0	0
ME1	0.53	4.13	0	0	0	1.28	4.62	0	0	0
OLIG2	17.97	4.1	3.40E-10	2.23E-07	1.25E-05	48.07	5.41	0	0	0
XAGE1B	6.71	-6.55	1.48E-07	5.79E-05	5.44E-03	5.9	-4.59	6.21E-06	2.19E-04	0.23
RP11-350E12.1	5.82	-9.53	2.00E-04	0.03	1	18.39	-28.66	1.11E-08	7.51E-07	4.05E-04

**Figure 4:**
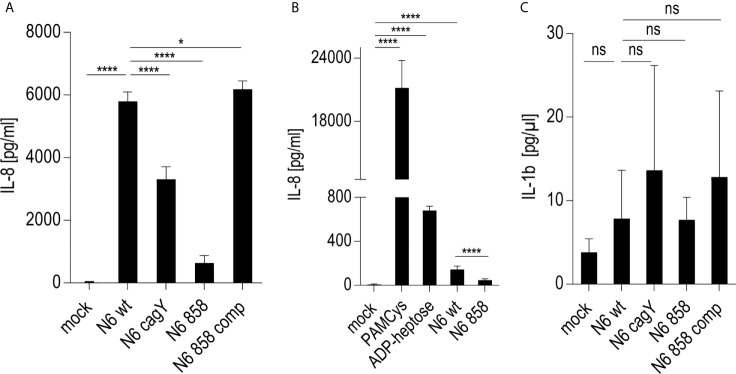
Response of NLRP3-deficient Thp-1 monocyte/macrophage cells (Thp-1 dNLRP3) to live *H. pylori* and pure ADP-heptose. **(A, B)** show IL-8 secretion by co-incubated, NLRP3-deficient Thp-1 cells. **(A)** Thp-1 NLRP3def cells co-incubated with live *H. pylori* bacteria of different genotypes at MOI 5 for 20 h in 24 well format **(B)** Thp-1 dNLRP3 cells co-incubated with *H. pylori* ETL (50 µl/well) from OD_600_ = 2 in 24-well plate for 20 h. As controls, for TLR (3) activation, PAMCys at 400 ng/well, and for ALPK1 activation, ADP-heptose at 5 µM, respectively, were added in parallel experimental conditions. **(C)** IL-1β secretion by NLRP3def Thp-1 cells co-incubated with live *H. pylori* variants as in **(A)**. Results of one representative experiment out of three independent experiments are shown in each panel. Cell responses in **(A–C)** were quantitated using cytokine ELISA. Cells were not primed before adding the respective stimuli. IL-8 was not decreased (rather increased) in NLRP3-deficient cells upon *H. pylori* co-incubation, while IL-1β was significantly decreased and lost the CagT4SS-and heptose-dependent phenotype upon deficiency of NLRP3. Statistical differences were calculated by unpaired student’s *t*-test. Significant p values: ****p < 0.0001; *p < 0.05; ns is non-significant.

In order to compare transcript regulation for a wider range of cell-co-incubated mutants, a selected panel of transcripts, mainly such involved in the innate immune response, collected from the comparison of global transcriptomes, were subsequently verified using cDNAs generated from Thp-1 cells co-incubated with various *H. pylori* mutants (live bacteria) (**Figures 3C–L**). Amounts of transcripts of downstream regulated cytokines IL-8, IL-1β; and CCL4 were comparatively determined by qPCR for the *cagY* mutant (HP0527; CagT4SS functionally deficient), and the heptose HP0858 (*hldE*) mutant in addition to cells co-incubated with the parental bacteria (**Figures 3C–E**). We determined significant differences between the conditions, in particular between wild type- and mock- or HP0858 (*hldE*) mutant-co-incubated cells (decrease of cytokine transcripts). Interestingly, in qPCRs, which test for transcript amounts of cytokine genes (il-1β and il-8) as a marker for transcription factor, e.g. NF-κB, activation, not for cytokine release, the *cagY* mutant presenting with a defective CagT4SS did not show reduced IL-8 or IL-1β transcript amounts in comparison to wild type, in contrast to cytokine release. In contrast, the ADP-heptose-deficient HP0858 mutant always displayed reduced transcript amounts for those specific cytokine transcripts. This data strongly indicates that for cytokine transcript induction in the presence of free heptose metabolites, no active T4SS is required (indicating active metabolite uptake), whereas for cytokine release by live bacteria (as shown by ELISA in **Figures 1, 2**), even when the bacteria actively produce heptoses, an active bacterial transport system, provided by the CagT4SS, is necessary to strongly promote cytokine release. Other activities by the CagT4SS may influence cell/caspase-1 activation for cytokine release. Deficiencies in cellular cytokine release induced by the heptose HP858(*hldE*) mutant bacteria in contrast to the wild type bacteria, as outlined in the previous paragraphs, are therefore likely to result rather from a reduction of induction of primary cytokine transcript than from a defect of the CagT4SS. Defects of the *H. pylori hldE* mutant in cytokine transcript upregulation were completely reverted by complementing the HP0858 gene *in trans* in another (*rdxA*) locus of the bacterial chromosome (17). Transcription factor NF-κB subunit transcript (NFKB2) was very strongly induced by both pure ADP-heptose and wild type *H. pylori*, but not by the *hldE* mutant in these settings, while TNFα transcript was not induced by ADP-heptose and not differently induced between parental wild type bacteria and *hldE*- bacteria. Comparative qPCR for a selection of genes coding for upstream receptors involved in heptose sensing and other innate immune pathways activated by *H. pylori* bacteria (NLRP3, ALPK1, TIFA, NOD1, TLR2; **Figures 3F–J** and **4**) revealed that transcripts for these innate immune pattern recognition receptors (PRR) were not substantially changed under any of the tested co-incubation conditions over mock.

**Figure 5:**
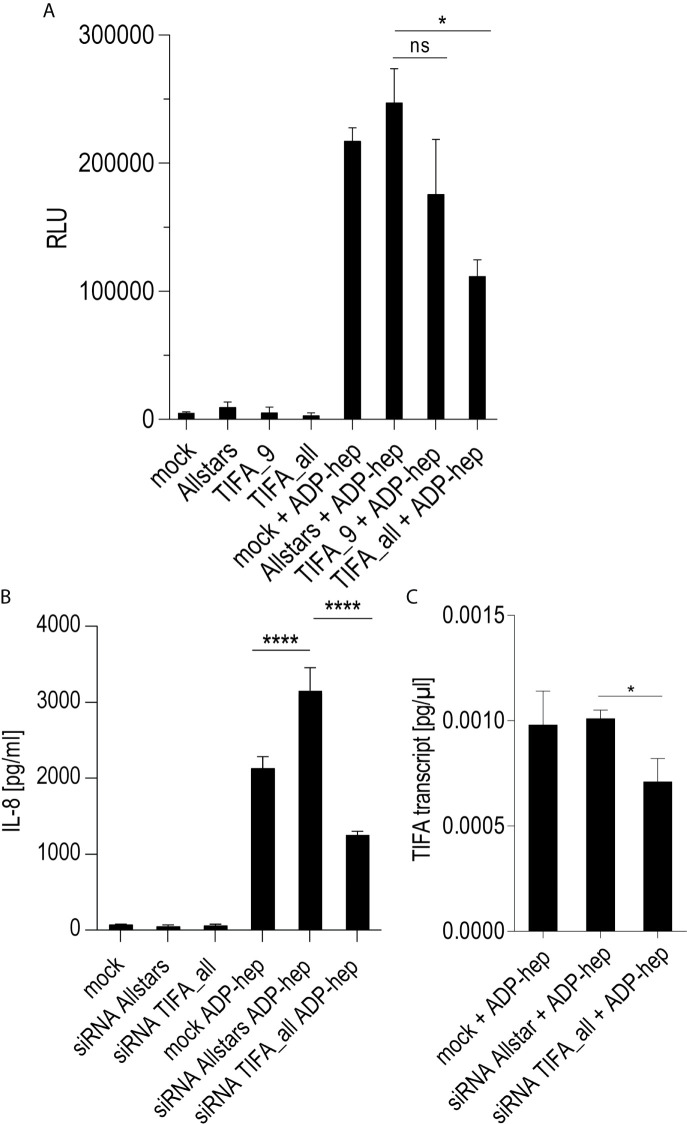
siRNA knock-down of TIFA reveals its important role in activation of Thp-1 cells by ADP-heptose. Panels **(A–C)** show the results of siRNA treatment of Thp-1 cells. **(A)** shows the quantitation of TIFA siRNA effects on NF-κB-dependent luciferase reporter activity (Thp1_luc) after stimulation with 2.5 µM pure ADP-heptose (samples with ADP-hep). Either one single siRNA (TIFA_9) was used, with a moderate but significant reducing effect on activation, and a combination of four siRNAs (see *Methods*) showed a strongly significant effect on NF-κB activation by pure ADP-heptose. Not heptose-stimulated controls of the mock-transfected or siRNA-treated cells are shown alongside (bars on left side), with very little detectable NF-κB activity. Mean and standard error of four independent biological replicates are depicted for each condition. Panel **(B)** shows the reduction of IL-8 release into cell supernatants upon TIFA siRNA knock-down (combination of four TIFA siRNAs) of Thp-1 cells treated with pure ADP-heptose (ADP-hep, 2.5 µM), in comparison to the Allstars negative control siRNA and a mock-transfected sample without siRNA. Same conditions incubated without ADP-heptose are shown on the left side, with no significant activation. Mean and standard errors of six replicates for each condition are shown. In **(C)**, the significant reduction of TIFA transcript in TIFA siRNA-transfected cells in comparison to Allstars siRNA-treated cells and mock-transfected cells (all under co-incubation conditions with ADP-heptose, 2.5 µM, ADP-hep) was verified by qPCR, normalized to human GAPDH transcript, in triplicate samples. Statistical differences were calculated by unpaired student’s *t*-test. Significant p values: ****p < 0.001; *p < 0.05; ns is non-significant.

Except for TLR2, which was always strongly active in Thp-1 cells (see PAMCys controls used in most assays), the above-mentioned PRR as well as NOD2, TLR4, and TLR9 were not highly activated in Thp-1 cells (**Supplementary Figures S4C, D**). Mining the transcriptomes for M1- and M2-macrophage-specific signatures of transcripts in the transcriptomes revealed that co-culture of the Thp-1 cells with either wild type *H. pylori* or free ADP-heptose seemed to emphasize the induction of M1-related transcripts (**Supplementary Figures S4A, B**). IFN-*γ* was not produced in the supernatants of heptose- or bacteria-co-incubated Thp-1 cells (data not shown), which suggested that JAK/STAT pathway or IRF transcription factors were not activated under the short-term co-incubation conditions used. Finally, the question needed to be answered whether the TIFA-ALPK1 pathway (21) is responsible for heptose-mediated activation in monocyte/macrophages. Using siRNA knock-down of TIFA, the activity of pure ADP-heptose on the cells was significantly reduced to more than half of the siRNA negative control (determined by luciferase reporter and IL-8 secretion; **Figures 5A, B**). At the same time, we were able to reduce TIFA transcript in difficult-to-transfect Thp-1 cells by about 30% (**Figure 5C**). These assays confirmed TIFA as an important mediator of responses against ADP-heptose in Thp-1 monocytic cells.

### Heptose-Dependent Activation of Cellular Signal Transduction by *H. pylori* in Monocyte/Macrophage-Like Cells Is Not Influenced by NLRP3

We next investigated whether one major intracellular innate immune sensor, NLRP3, a central inflammasome activator which has been characterized to be strongly activated in macrophages by *H. pylori* (14, 15), contributes to or interferes with the early heptose-dependent signaling and cytokine release observed in professional phagocytes, as we reported above. This approach was also designed to test whether heptose metabolites are involved in the NLRP3-dependent response, and whether NLRP3 can feed back into or synergize directly with the (ADP-)heptose-dependent signaling pathway. For this purpose, we utilized a NLRP3-deficient (NLRP3-def) Thp1 cell line in comparison to the Thp-1 wild type macrophage line. When we co-cultured the NLRP3-def cells with *H. pylori*, we observed, as expected, only a very low response of inflammasome-dependent IL-1β (cytokine release and transcript response; **Figure 4**), to live bacteria co-incubation at different time points, in comparison to the wild type, NLRP3-competent, cells. The heptose-deficient and *cag*PAI mutants did not show a significant difference in IL-1β release on the NLRP3-def cells compared to the parental wild type bacteria. In the quantitative IL-8 assays, overall cytokine release was even considerably stronger in the NLRP3-deficient cells than in the NLRP3-competent Thp-1 parent, for co-incubation with live bacteria, or in parallel experiments with ETLs of the same strains. This result indicated no activating role of NLRP3 in promoting the IL-8 output triggered by *H. pylori* in this cell type. Interestingly, the *H. pylori* HP858/*hldE* mutant also activated the NLRP3-deficient cells to produce IL-8, albeit lower than for wild type bacteria, but very likely higher than in Thp-1 parental cells. Even if a direct comparison between two different separately co-incubated cell lines is not precise, we determined that pure ADP-heptose showed roughly the same absolute activation levels in NLRP3-deficient Thp-1 cells as in parental Thp-1. These results suggest that, while TIFA, as a response adaptor to heptose metabolites, can increase NLRP3 responses as reported in previous studies (66), lack of NLRP3 has a limited influence on ALPK1-TIFA signaling by ADP-heptose or by *H. pylori*, in particular on the outcome of IL-8 release and NF-κB activation in these macrophage-like cells. The LPS heptose recognition pathway by ALPK1-TIFA seems therefore not to be positively or negatively influenced by NLRP3.

## Discussion

The aim of this study was to investigate the role of LPS heptose-mediated signaling by *H. pylori* in human monocyte-like cells, which can be differentiated to a macrophage-like phenotype, exemplified by the model cell line Thp-1 (47, 53, 67). The recently discovered heptose-dependent innate recognition and signaling process (68) had not been studied specifically in human phagocytic cells. We and others have recently demonstrated that human epithelial cells are selectively activated at early time points by Gram-negative bacteria through heptose metabolites of the LPS inner core biosynthesis, which mediate intracellular activation *via* the ALPK1-TIFA axis (17, 18, 59, 68, 69).

In epithelial cells co-incubated with *H. pylori*, this TIFA- and ALPK1-mediated signaling process is CagT4SS-dependent and LPS heptose metabolite-dependent (16, 17). More recently, further biochemical analyses have refined that the bacterial LPS heptose metabolite that is predominantly and directly recognized by the cellular kinase ALPK1 seems to be predominantly the activated inner core LPS metabolite ADP-heptose (21, 22, 70). ADP-heptose, in *H. pylori* and other Gram-negative bacteria, is specifically generated by the second biosynthesis step catalyzed by the bifunctional enzyme RfaE/HldE (HP0858 in *H. pylori* strain 26695) (17, 21). For *H. pylori*, ADP-heptose or potentially other heptose metabolites are transported into human epithelial cells by the CagT4SS (17). So far, it has been established that monocytic cells or activated macrophages isolated from mice seem to recognize *H. pylori* mainly *via* TLR2, TLR4, TLR9, NOD2, and NLRP3 activation (11, 14, 15). In addition, previous published work has also found a contribution of the CagT4SS in the early activation (TLR independent) of macrophage-like cells, exemplified by results collected for Myd88-Trif-deficient primary bone-marrow-derived mouse macrophages (44). In this prior work, the authors described that anergic, broadly TLR signaling-deficient mutant macrophages recognized the *H. pylori* CagT4SS by innate immune activation and that this was a rather NOD1/NOD2- and Rip2-independent process, although the authors could not attribute it to a precise mechanism (44). In the light of results from the present study, it now seems very likely that the activation mechanism described by Koch and colleagues is driven mainly by heptose metabolites, presumably ADP-heptose.

In our present work, we tested the activation signature by *H. pylori* and purified bacterial ADP-heptose using the Thp-1 human monocyte-macrophage-like cell line (naïve or pre-differentiated). *In vivo*, professional phagocytes can come into close contact with *H. pylori* in the stomach mucosa and induce cytokine production, inflammation, and induced neutrophil immigration (71) when exposed to the bacteria. In our present study, non-pre-activated Thp-1 cells differentiated upon *H. pylori* or free ADP-heptose co-incubation into a macrophage-like, adherent cell phenotype, a process, which was starting around 8 h after the initiation of the co-incubation. At later time points post-differentiation and in an MOI-dependent manner, the cells started to produce and release IL-8 and IL-1β. This response was indeed significantly different whether *H. pylori* wild type or heptose mutants or *cag*PAI mutants were co-incubated. Hence, in this setting, LPS heptose metabolite production, partially aided by translocation of the metabolites into the Thp-1 cells *via* the T4SS, was a major driver of cytokine (IL-8) release and NF-κB activation, but not of IFN-*γ* release. Heptose-dependent cell activation by pure ADP-heptose and *H. pylori* bacteria was also confirmed for primary human monocytes and monocyte-derived macrophages, but the details need further clarification. The relative contribution of the active CagT4SS was not as strong as in co-incubation experiments with human gastric epithelial cells (16, 17), and also correlated with the bacterial MOI used. CagA was not involved in the activation of Thp-1 cells in our present settings, but an active CagT4SS was nevertheless important. Previous results gathered in mouse macrophages (44) did not emphasize an important role of the CagT4SS in macrophage activation. However, in addition to potential differences between mouse and human macrophages in response to heptose metabolites, which needs to be tested in future studies, this divergent result from our study may also be explained by the use of different *H. pylori* strains or of higher MOIs (50 bacteria per cell) in their study as opposed to rather low MOIs which we were testing here (5 to 25 bacteria per cell). Congruently with their study in mouse cells (44), and with the characterization of human macrophages in *H. pylori*-infected patients (72), we collected some evidence that *H. pylori* differentiates human monocyte-like cells towards a proinflammatory M1 macrophage phenotype, or a hybrid M1/M2 phenotype (62–64), which was partially dependent on bacterial heptose biosynthesis. IRF8 transcript upregulation, a marker for macrophage maturation, was also induced here by heptose coincubation. Phagocytosis during short-term exposure of Thp-1 cells up to 24 h was not affected by the presence of pure heptose metabolite. By knocking down TIFA in monocytic Thp-1 cells, we confirmed the important role of TIFA in response to heptose metabolites in this cell type.

Using bacterial mutants, purified bacterial lysates and pure ADP heptose, we determined for the first time that the bacterial LPS inner core metabolite ADP-heptose can be taken up by human monocyte/macrophages in the absence of a dedicated bacterial secretion/injection system. Comprehensive transcript analyses demonstrated that a human gene encoding a monosaccharide transporter of unknown specificity, SLC2A6 (Glut6), which seems to be highly relevant in macrophages (73), was strongly upregulated by heptose exposure. This result might earmark SLC2A6 as a potential importer of heptoses in macrophage-like cells. Interestingly, SLC2A6 was reported recently to be an upregulation marker for the M1 polarization of macrophages (74). In a similar manner as pure ADP-heptose, ETLs generated from *H. pylori* wild type strain and isogenic CagT4SS-deficient mutants activated the Thp-1 cells, as long as ADP-heptose biosynthesis was intact. Cytochalasin D treatment did not reduce the activation by and uptake of pure metabolite to a major extent; this finding supports the possibility of an active transport/import mechanism for heptose metabolites into the cells, as opposed to a primarily random phagocytic mechanism of uptake. ETLs generated from *H. pylori* ADP-heptose biosynthesis deficient mutants (*hldE*), in contrast, were much less able to induce IL-8 secretion, or transcript, or NF-κB activation, in Thp-1 cells. Conditioned bacterial growth media from ADP-heptose proficient bacteria, with or without a functional CagT4SS, were also able, albeit to a lesser extent, to induce monocyte/macrophage IL-8 production. This result indicates that *H. pylori* grown in the absence of cells seem to release only low amounts of heptose metabolites.

Our genome-wide transcript analyses revealed a strong influence of pure ADP-heptose on the global human transcriptome of Thp-1 cells. Signature transcripts of Thp-1 activated by ADP-heptose at early time points encompassed il-8 (cxcl8), ccl2, ccl3, ccl4, and il-1β, but not il-6 or il-10. Central activation markers of both, ADP-heptose and *H. pylori* wild type bacteria, comprised increased transcript amounts of genes coding for NF-κB transcription factors and coactivators, complement factors, and genes involved in intracellular recognition of double-stranded RNA of the OAS family (65). We detected a substantial overlap of about 350 transcripts regulated by pure ADP-heptose or live *H. pylori* wild type bacteria. Transcription factor NF-κB gene transcript (NFKB2) was very strongly induced by both ADP-heptose and wild type *H. pylori*, but less so by the *hldE* mutant in these settings. In addition, subsets of specific transcripts were differentially regulated by free ADP-heptose or live *H. pylori*. One of the most striking differences was the downregulation of PYCARD/ASC transcript for live *H. pylori* as compared to mock, and the upregulation of the same transcript for the ADP-heptose-treated cells over mock-treated. This effect and transcripts in the same cluster of regulation merit to be followed up in future work. In direct comparisons, live *H. pylori* wild type bacteria and isogenic LPS heptose mutants were rather distinct in their activation of transcripts, although both also showed some transcript overlap.

Despite the identified differences, we determined that transcriptional regulation and NF-κB activation overlapped between Thp-1 cells co-incubated with ADP-heptose, *H. pylori* wild type bacteria or isogenic *hldE* mutants. Since TLR2 was confirmed to be highly expressed in these phagocytic cells and the canonical TLR2 ligand PAM3Cys-SK4, used as a control stimulus in our experiments, activated the cells very strongly, we assume that TLR2 ligands present in the *hldE* mutant bacteria and its lysate preparations contribute to the observed signaling overlap, *via* the TLR pathway. Co-culture with *H. pylori* and *H. pylori*-isolated DNA can activate neutrophil IL-8 production *via* TLR9 (11). However, we did not see a substantial activation of the non-pre-primed Thp-1 cells by *H. pylori* DNA, or by NOD1, NOD2 ligands. Therefore, we surmise that TLR9, NOD1, and NOD2 signaling are rather weak in these settings. NOX production was not observed in Thp-1 cells under the conditions and settings used in the present study (own unpublished data) and was therefore not considered to be affected by heptose. NLRP3 activity seemed to play a minor or no role in the heptose-mediated innate cell activation of these monocyte/macrophages. Earlier work reported that soluble molecules extracted from *H. pylori* are able to activate phagocytic cells to produce IL-8 (14). This seems to be in tune with our present results that heptose production and treated lysates of *H. pylori* bacteria enriched in metabolites can induce proinflammatory signaling, NF-κB activation and IL-8 secretion in Thp-1, and human primary monocytes and monocyte-derived macrophages, very likely *via* the ALPK1-TIFA axis. This might be partially supported by phagocytic or other uptake activities that those cell types exhibit, since cytochalasin D treatment partially inhibited the activation.

Other cells that should be studied for the influence of *H. pylori* heptose-ALPK1-TIFA signaling in the context of *H. pylori* are indeed neutrophils and dendritic cells, which infiltrate the gastric tissue during human *H. pylori* infection (75). In *H. pylori*-infected neutrophils, NLRP3 was previously established as pattern recognition receptor with a major influence (76). Others have reported that CagT4SS-impaired wild type strains and specific CagT4SS mutants (VirB4, VirD4) induced somewhat higher amounts of IL-1β, and less IL-10 in neutrophils than CagT4SS-competent wild type bacteria (77), which might indicate a different role of heptose signaling in those cells.

Taken together, *H. pylori* activates phagocytic cells/macrophages at early time points in a specific manner. This effect is primarily *cag*PAI- and heptose-dependent, if naïve monocyte-like cells come into contact with *H. pylori*. This might apply to the natural habitat in the stomach, where *H. pylori*-naïve monocytes or other phagocytic cells may immigrate or reside in the local tissues. LPS inner core heptose metabolites, most likely including ADP-heptose (21), can be one major activating factor for such early activation and priming *in vivo*. Pure ADP-heptose exerted a comparable effect as wild type bacteria in our assays, both in time course and in strength. For macrophages in direct contact with live *H. pylori* bacteria, activation relied on metabolite transport by an active CagT4SS, which can be provided only by live bacteria. Upon release of heptose metabolites (ADP-heptose), which can occur by different means such as by spontaneous bacterial lysis *in vivo*, induced lysis, or phagocytosis, monocyte/macrophages are also able to take up heptose metabolite without the activity of the CagT4SS or live bacteria. Very likely, other Gram-negative pathogenic bacteria invading different body sites, such as the respiratory, reproductive or intestinal tract, can also activate phagocytic cells by LPS heptose metabolites, in the presence or absence of dedicated bacterial secretion systems. Remaining questions, for example with regard to the role of these interactions and responses for the chronic inflammation and disease settings *in vivo*, or for the interaction with other professional phagocytes and antigen-presenting cells are important subjects to be investigated in further work. A prophylactic and therapeutic vaccine would be a much-desired tool to combat *H. pylori*-mediated diseases and cancerogenesis, but its successful design seems to be continuously hampered by *H. pylori* immune evasive and modulatory mechanisms. For these reasons, in depth studies about the specific interaction of *H. pylori* with various phagocytic cell types are urgently needed.

## Data Availability Statement

The datasets presented in this study can be found in online repositories. The names of the repository/repositories and accession number(s) can be found below: NCBI, PRJNA685657.

## Author Contribution

LF and SCS contributed to the design of the study, performed and interpreted experiments, and co-authored the paper. MH, MG and MA performed and interpreted experiments and provided materials. CJ conceived the study, performed and interpreted experiments, acquired funding, and wrote the paper. All authors contributed to the article and approved the submitted version.

## Conflict of Interest

The authors declare that the research was conducted in the absence of any commercial or financial relationships that could be construed as a potential conflict of interest.
