# Supplementary material for: Contribution of Heptose Metabolites and the cag Pathogenicity Island to the Activation of Monocytes/Macrophages by Helicobacter pylori
Source: Front Immunol. 2021 May 19;12:632154. doi: 10.3389/fimmu.2021.632154 (PMC8174060; doi:10.3389/fimmu.2021.632154)
Supplement: Supplementary file 3 [file Presentation_1.pdf]

Supplementary Information
Supplementary Figures

Contribution of heptose metabolites and the *cag* Pathogenicity Island to the activation of monocytes/macrophages by *Helicobacter pylori*

L. Faass, S.C. Stein, M. Hauke, M. Gapp, M. Albanese & C. Josenhans

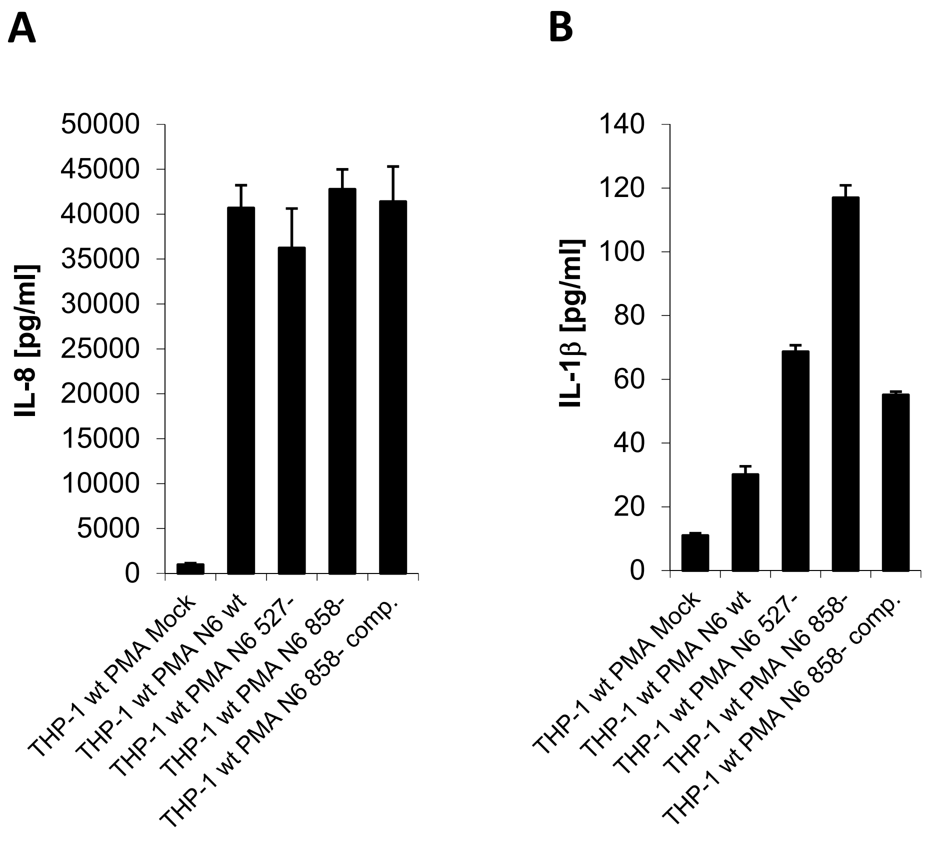

Fig. S1: heptose biosynthesis and the CagT4SS have no direct role in the capacity of live *H. pylori* to induce IL-8 or IL-1β secretion by PMA-preactivated human monocyte/macrophage cell Thp-1. Thp-1 cells which were primed and pre-activated by PMA (see methods), and only afterwards co-incubated with live *H. pylori* (post co-incubation for 4 h). Cytokines in the cell supernatants were quantitated by ELISA **A)** IL-8 secretion; **B)** IL-1β secretion. MOI was set at 25 bacteria per cell. In addition to parental wild type bacteria of strain N6, *cagY* (527-, T4SS functioanlly deficient), *hldE* (858- , core heptose biosynthesis-deficient), and *hldE*-complemented bacteria (858- comp.) were tested.

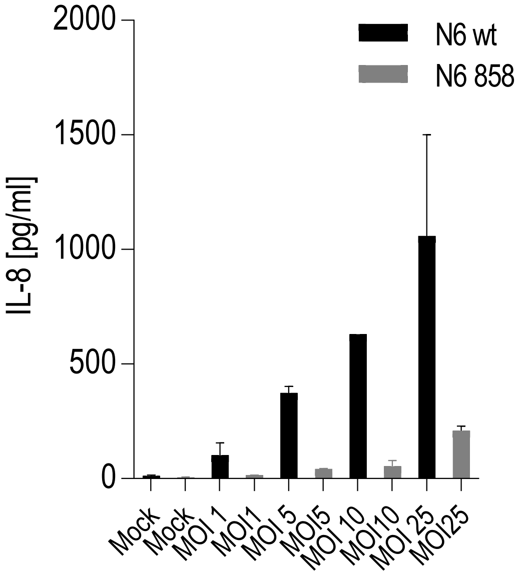

Fig. S2: cell activation in monocyte-like Thp-1 cells upon co-incubation with live *H. pylori* bacteria are influenced by bacterial MOI. Thp-1 cells were co-incubated with *H. pylori* N6 wild type N6 bacteria or isogenic *hldE* mutant bacteria at different MOIs; 20 h post-co-incubation, IL-8 cytokine secretion into the supernatants was determined by ELISA.

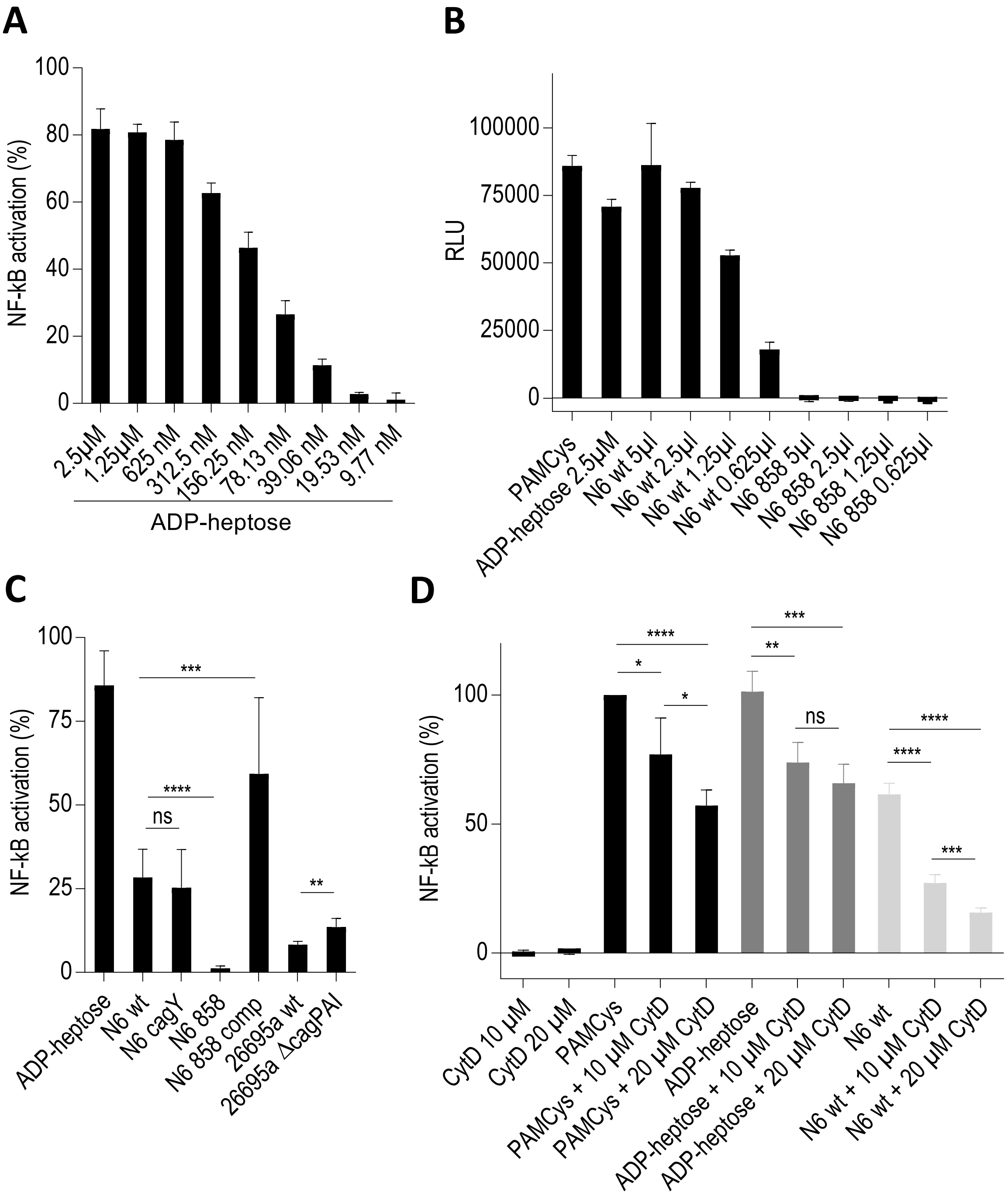

Fig. S3: Characteristics of NF-κB activation in Thp-1 cells by pure ADP-heptose, *H. pylori* enzyme-treated lysates (ETL) or bacterial culture supernatants. A) concentration-dependent activation of NF-κB in Thp1_luc reporter cells by pure ADP-heptose. ADP-heptose was co-incubated with the cells at 5 µM for 4 h. For each concentration, NF-κB activation in %, relative to a PAMCys control (PAMCys) which was set to 100%, is depicted. **B)** Metabolite-enriched ETLs generated from *H. pylori* N6 wild type strain and its isogenic *hldE* mutant (at different volumes as indicated, 96 well plates) were applied to Thp1_luc reporter cells for 4 h. Arbitrary luminescence units (RLU) are shown for each condition. **C)** Culture **s**upernatants generated from liquid cultures of two different *H. pylori* strains as indicated and respective isogenic mutants in the *cag*PAI or heptose biosynthesis (*hldE*-, 858), (20 µl of supernatants per well in 96-well plates) were co-incubated with Thp1_luc reporter cells for 4 h (for strain descriptions see Table 1). Quantitation of luciferase activity is shown for each condition in percent of the positive control PAMCys (20 ng/50 µl), which was set to 100%. **D)** role of cytochalasin D (cytoskeleton inhibitor) in activation of Thp-1 cells (NF-κB) by pure ADP-heptose and live *H. pylori* bacteria. Thp1_luc reporter cells were co-incubated with pure ADP-heptose or live bacteria (strain N6, MOI of 5 bacteria per cell) for 4 h, in the presence or absence of the cytoskeleton inhibitor cytochalasin D (CytD). All values in D) are depicted in % of the positive reference (PAMCys, 100%). Statistical differences in **C**) and **D**) were calculated by unpaired student's *t*-test. Significant p values: ****p < 0.0001; ***p < 0.001; **p < 0.01; *p < 0.05; ns is non significant. Mock values (very low) were subtracted as background in all assays from A) through D) and are therefore not depicted.

Faass, Stein et al., Suppl. Fig. S3

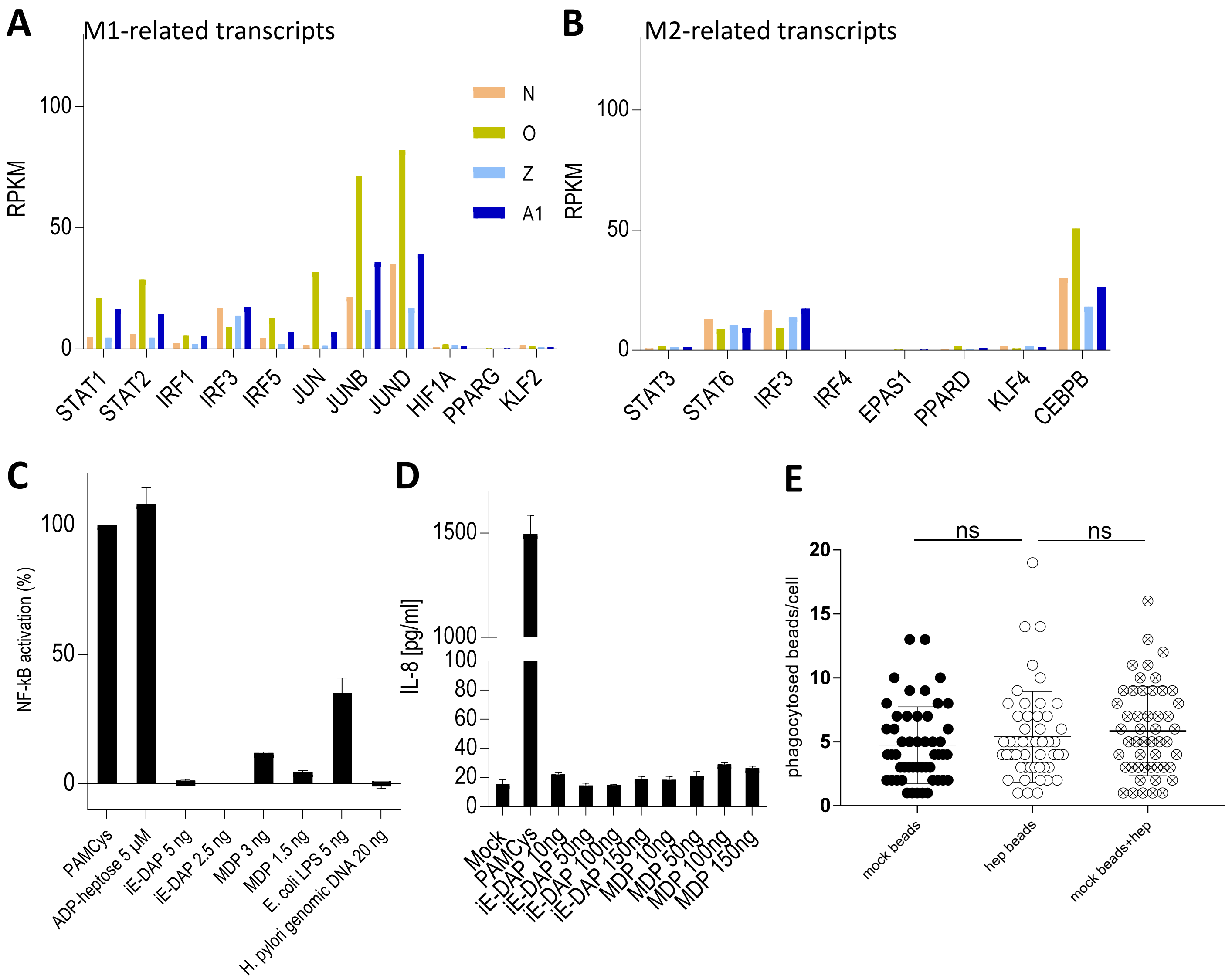

Fig. S4: Selected transcriptome results of Thp-1 cells for macrophage phenotype and contribution of various pattern recognition receptors and their ligands to Thp-1 activation and phagocytosis. assessing the presence and differential expression of transcripts involved in macrophage polarization. In panels **A**) and **B**), we assessed subpanels of transcripts (RPKM) involved in M1 (panel **A**) or M2 (panel **B**) macrophage polarization for differential expression in our comprehensive transcriptome datasets. N, O, Z, A1 designate mock1, *H. pylori* N6 wild type, mock2 and pure ADP-heptose co-incubation conditions with Thp-1 cells, respectively. Compare also Table 3. main Figure 3, and supplementary tables for transcriptome results. Detailed methods for transcriptome results and analyses can be found in the Methods' description. **C**) and D) testing for the activity of NOD1, NOD2, TLR4 and TLR9 ligands in comparison with PAMCys (positive control for activation) and ADP-heptose for NF-κB activation (**C**) or IL-8 secretion (**D)** in Thp-1 cells. In **C**), relative luminescence in % of the PAMCys control is depicted for NF-κB-dependent luciferase activation of Thp1_luc reporter cells (96 well, co-incubation for 4 h) are shown; mock values were subtracted as background). In **D**), IL-8 secretion into the supernatants of co-incubated Thp-1 cells (20 h p.c.) was quantitated by ELISA. PAMCys was applied in C), and D) as a control condition for NF-κB activation. **E**) phagocytosis by Thp-1 cells, co-incubated with fluorescent microbeads (1 µm diameter, $4x10^6$ beads/well in 24 well plate – 20 beads per cell) in the absence or presence of the MAMP ADP-heptose (at 2.5 µM). Ingested beads per cell were counted in fluorescence microscopy. Mock beads: Thp-1 cells co-incubated with beads for 4 h in the absence of innate stimulus; hep beads: Thp-1 pre-incubated with pure ADP-heptose for 16 h, then microbeads were added and co-incubated for another 4 h; mock beads+hep: Thp-1 cells were co-incubated with microbeads and ADP-heptose for 4 h. 50 cells were counted for each condition. Pairwise and multiple comparisons of statistically significant differences in bead uptake (shown on the y-axis) were performed using two-way ANOVA. ns is non significant.

Faass, Stein et al., Suppl. Fig. S4

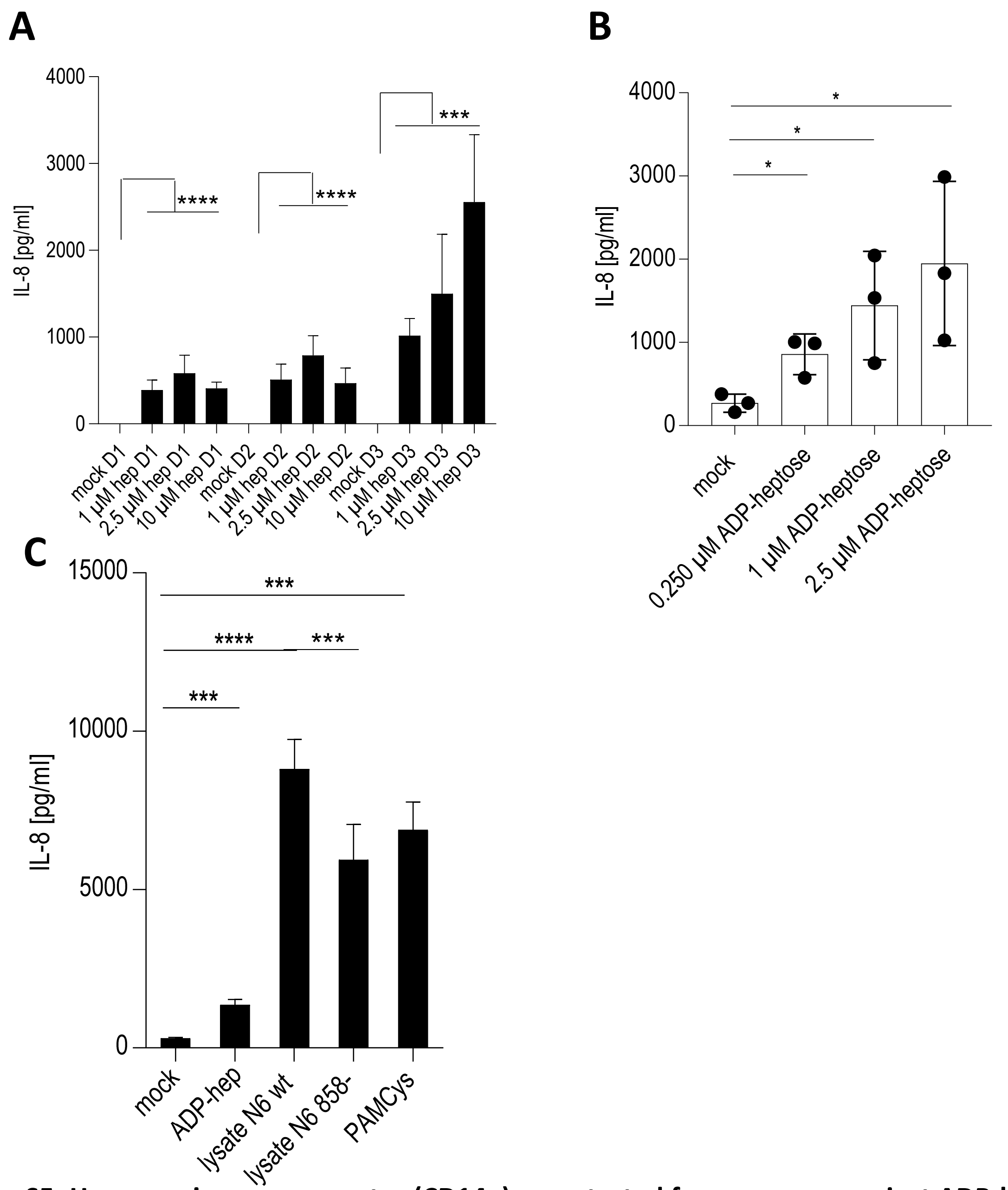

Fig. S5: Human primary monocytes (CD14+) were tested for responses against ADP-heptose and *H. pylori* bacteria. CD14+ cells were isolated and either co-incubated directly with ADP-heptose (hep) or differentiated to hMDMs, subsequently co-incubated with ADP-heptose or *H. pylori* enzyme-treated lysates **A**) Response of non-differentiated CD14+ human primary monocytes (2 x 10^5 cells/well in 24-well plate) from blood PBMC after exposure to pure ADP-heptose (hep) at indicated concentrations, for 20 h. CD14+ monocytes from three independent individual healthy donors (D1, D2, D3) were investigated. **B**) Concentration-dependent activation of differentiated CD14+ human primary monocyte-derived macrophages (2x 10^5 cells/well in 24-well plate) from three independent donors by pure ADP-heptose; All donors are combined in one bar for each condition; mean and standard error for the independent donors are shown for each condition. Despite some between-donor variation (see black ball-shaped symbols), the concentration-dependent activation is clearly visible and significant. **C**) Response of differentiated primary monocyte-derived macrophages (one donor) towards ADP-heptose (ADP-hep, 2.5 µM) or bacterial treated lysates enriched in small metabolites (50 µl pf ETL per ml cell medium in 24 well plates; co-incubation time 4 h). N6 is wild type of *H. pylori* strain N6; 858- is the isogenic *hldE* mutant of strain N6. PAMCys (400 ng/ml) was used as a positive control condition for cell activation. IL-8 secretion into the cell supernatants in **A**), **B**) and **C**) was quantitated by ELISA. Statistically significant differences between mock- and ADP-heptose- or bacteria-co-incubated cells were calculated using two-way ANOVA and are annotated as significant differences to the mock condition (in **A** and **B**) or differences between different co-incubation conditions in **C**); ***p < 0.001, ****p < 0.0001.

Faass, Stein et al., Suppl. Fig. S5

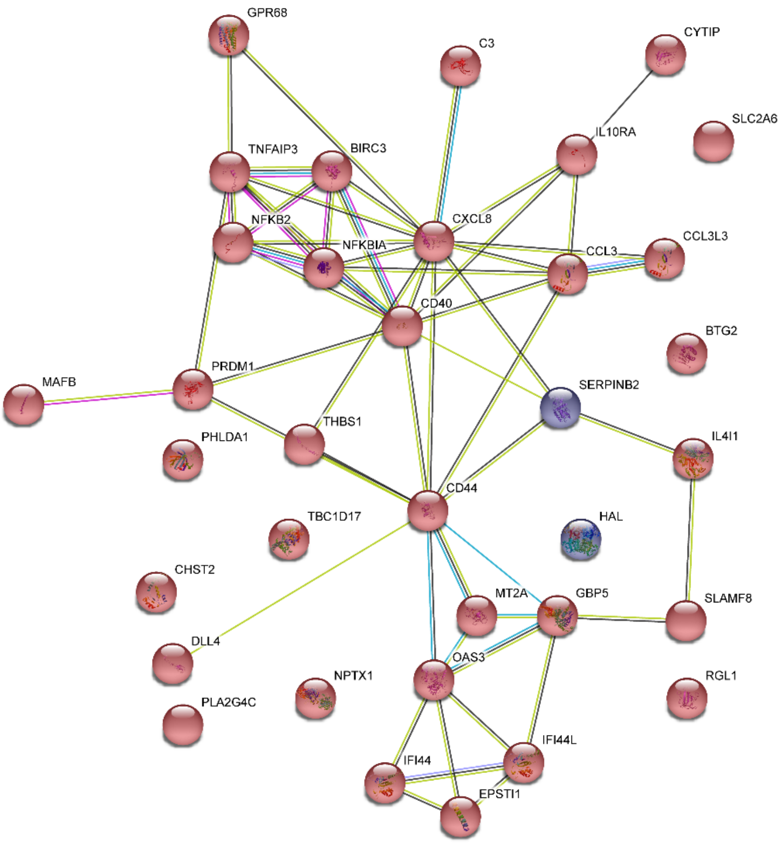

Fig. S6: Results of transcriptome sequencing: pathway analysis and visualization by STRING of transcripts differentially regulated in Thp-1 cells co-incubated with *H. pylori* N6 wild type bacteria or with isogenic *hldE* mutant bacteria. Comprehensive transcriptomes generated from mock-co-incubated and bacteria-co-incubated Thp-1 cells were compared with each other for differential transcript regulation. Subsequently the overlap of differential transcript regulation between wild type bacteria vs. mock (control condition) and *hldE*-mutant bacteria versus mock, respectively, was determined (see Venn digaram in main Fig. 3), and the genes in the intersection of both RNA-seq pairings were analyzed by STRING (stringent cut-off of 8-fold regulated). Commonly upregulated transcripts by the two conditions are shown colored in red, commonly downregulated transcripts are colored in blue. Upregulated genes show a substantial overlap between paired conditions. Some central nodes of activation correspond to main Fig. 3 panel O (STRING diagram), shown for cell incubation with pure heptose versus mock-co-incubated and related to NF-kB activation. Full results are contained in Supplementary Table S2.
